# Accurate molecular weight determination of small molecules *via* DOSY-NMR by using external calibration curves with normalized diffusion coefficients†
†In memory to Professor Paul von Ragué Schleyer, the protagonist of organolithium chemistry.
‡
‡Electronic supplementary information (ESI) available: Includes detailed information about the calibration curves, the model compounds and the calculation of the molar van der Waals-density. See DOI: 10.1039/c5sc00670h



**DOI:** 10.1039/c5sc00670h

**Published:** 2015-03-19

**Authors:** Roman Neufeld, Dietmar Stalke

**Affiliations:** a Institut für Anorganische Chemie , Georg-August-Universität , Tammannstrasse 4 , Göttingen , Germany . Email: dstalke@chemie.uni-goettingen.de

## Abstract

Determination of the aggregation and solvation numbers of organometallic complexes in solution is an important task to increase insight in reaction mechanisms. Thus knowing which aggregates are formed during a reaction is of high interest to develop better selectivity and higher yields. Diffusion-ordered spectroscopy (DOSY), which separates NMR signals according to the diffusion coefficient, finds increasing use to identify species in solution. However, there still is no simple relationship between diffusion coefficient and molecular weight (MW). Some methods have been developed to estimate the MW but still with a significant error of ±30%. Here we describe a novel development of MW-determination by using an external calibration curve (ECC) approach with normalized diffusion coefficients. Taking the shape of the molecules into account enables accurate MW-predictions with a maximum error of smaller than ±9%. Moreover we show that the addition of multiple internal references is dispensable. One internal reference (that also can be the solvent) is sufficient. If the solvent signal is not accessible, 16 other internal standards (aliphatics and aromatics) are available to avoid signal overlapping problems and provide flexible choice of analytes. This method is independent of NMR-device properties and diversities in temperature or viscosity and offers an easy and robust method to determine accurate MWs in solution.

## Abstract 2

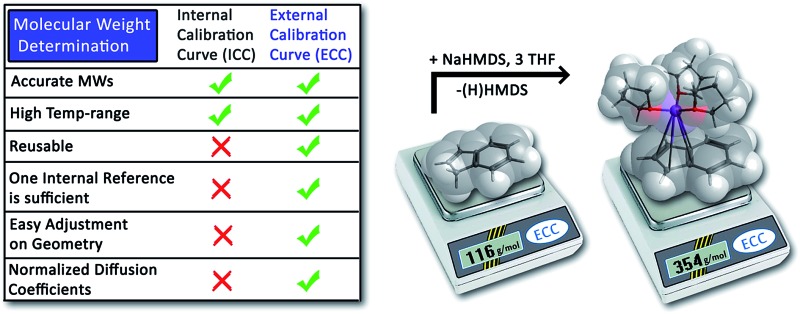
We describe a novel development of MW-determination by using an external calibration curve approach with normalized diffusion coefficients.

## Introduction

Chemists have always had a vital interest in the size of molecules. Especially the aggregation and solvation numbers of organometallic intermediates play a dominant role in reaction mechanisms and product forming.^1^ Therefore the knowledge of reactive aggregates is a prerequisite to understand how molecules react and why they form which products.^2^ Apart from colligative property measurements, such as cryoscopy,^3^ diffusion-ordered spectroscopy (DOSY) has gained increasing importance in this area.^4^ The physical observable that can be derived from the diffusion NMR experiment is the diffusion coefficient *D* that is sensitive to size and shape of the molecular species.^5^ A number of empirical methods for relating diffusion coefficients to the molecular weight (MW) have been proposed.^6^ Often the Stokes–Einstein equation^7^ and its modifications^8^ are useful and enable molecular size estimation of large particles that are much larger than the solvent.^9^ Besides that, especially the empirically derived power law (eqn (1)) is probably the most powerful class of relations which correlates the MW and the diffusion coefficient according to

1 *D* = *K*MW^*α*^

This power law^10^ gives good results but is restricted to a specific class of compounds.^11^ Especially the polymer community applied it to estimate the MW distribution of polymer solutions like *e.g.* globular proteins,^11^ oligosaccharides,^12^ polyethyleneoxides^13^ and denatured peptides^14^ in variable solvents. Even small molecules correlate to the power law, as demonstrated in the work of Crutchfield and Harris.^15^ Unfortunately this work only allows a MW estimation with a relatively high error of ±30%. For small organometallic molecules Li and Williard *et al.* have used an analogue approach of the power law by introducing at least three internal references to one NMR sample in order to get an internal calibration curve (ICC).^16^ For small molecules this method gives much better results. They were able to characterize for example THF-solvated LDA to be dimeric^17^ and a 1 : 1 mixture of LiHMDS with HMPA (hexamethylphosphoramide) to adopt a disolvated dimeric structure in TOL-*d*_8_ solution.^18^ The same ICC-method was employed by Armstrong and Mulvey *et al.* to characterize diisopropylamide and 2,2,6,6-tetramethylpiperidide (TMP) turbo-Grignard reagents in THF solution.^19^ Unfortunately the ICC-method has some important disadvantages: on the one hand the ICC employs just a few references (mostly 3) and is often based on a small molecular weight distribution. On the other hand each ICC is only useful for one NMR sample. Additionally all of the internal references are limited to a lot of prerequisites: (a) they should be inert to the analyte in solution; (b) the NMR signals should not overlap with other components; (c) the internal references should have no coordinating ability to the analyte; (d) they should be well soluble in the solvent and finally (e) the internal references should have a well set molecular weight distribution.^16^ Therefore it is a big challenge to choose the appropriate internal references, because often at least one of the above-mentioned exigencies would not be met.

In this article we describe the use of an external calibration curve (ECC) approach with fixed diffusion coefficients. This method allows highly accurate MW-determination without the necessity of multiple references that disturb the measurements. Furthermore we show for the first time how solvent temperature, concentration, the shape and the mass density of analytes influence the power law derived MW-determination. And finally we use ECCs to determine the aggregation of Na-indenide in THF-*d*_8_ solution.

## Results and discussions

### Application of a normalized reference system with fixed diffusion coefficients

**Fig. 1:**
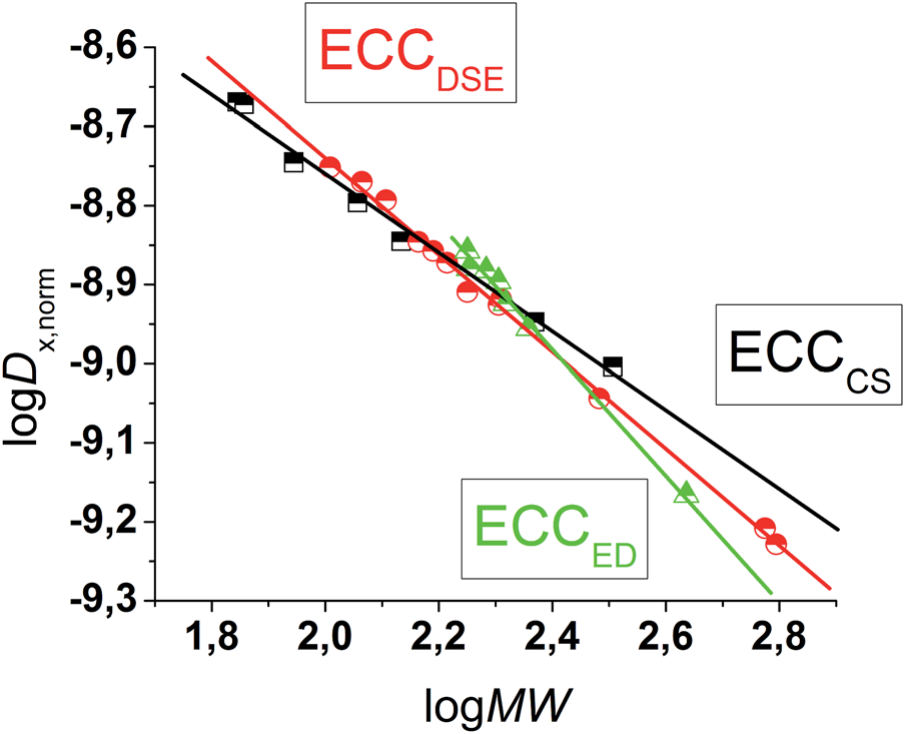
log *D versus* log MW in TOL-*d*_8_. All compounds were normalized to log *D*_ref,fix_(ADAM) = –8.8454, see Table 4.

DOSY spectra are frequently affected by various sources of errors like *e.g.* diversities in temperature, fluctuation, convection, viscosity and concentration effects. Additionally the NMR-device constants like *e.g.* gradient strength and pulse duration influence the absolute diffusion coefficient. So it becomes clear that DOSYs only can be compared under *ceteris paribus* conditions. To overcome the complications of these effects and to enable tabulated diffusion coefficients, the use of an internal standard is necessary. Those standards provide more resilient diffusion coefficients for any changes in the NMR sample.^20^ This ratio, often termed as relative diffusivity *D*_rel_ is defined as:

2 *D*_rel_ = *D*_x_/*D*_ref_

where ref and x correspond to the reference and analyte, respectively. This approach reduces the impact of viscosity and temperature and provides more robust data.^21^ Besides the above-mentioned advantages this method has a disadvantage. Eqn (2) produces relative diffusion values that always depend on the one reference molecule it has been referenced for. This reference has no strict or defined diffusion value. We found that it is advantageous to employ relative diffusion coefficients with fixed diffusion values. Subsequently we show that this approach allows not only one molecule to act as a reference rather than every compound that is part of the calibration curve to act as an internal reference. We realized that the logarithmic diffusion values are connected approximately linearly, independent of gradient and magnetic field strength, gyromagnetic ratio, gradient pulse duration, and changes in temperature or viscosity. That is why the linear eqn (3) was empirically derived:

3 log *D*_x,norm_ = log *D*_ref,fix_ – log *D*_ref_ + log *D*_x_

where log *D*_ref,fix_ is the fixed value of the reference, log *D*_ref_ the measured diffusion coefficient of the reference, log *D*_x_ the diffusion coefficient of analyte x and log *D*_x,norm_ the relative diffusion value of the analyte x normalized to the reference. Eqn (3) ensures that all molecules get a normalized diffusion coefficient relative to the internal reference. For our measurements we picked for all TOL-*d*_8_ solvates adamantane (ADAM) and for all THF-*d*_8_ solvates 2,2,3,3-tetramethylbutane (TMB) as internal references (see Table 1). The validity of eqn (3) was proved by measuring DOSY spectra of various model compounds on two different NMR spectrometer devices, where one spectrometer had calibrated and the other uncalibrated gradients. Although differences in gradient calibration, temperature and concentration automatically give deviations in absolute diffusion (see ESI, S-Fig. 1‡), eqn (3) provides excellent results for the normalized diffusion coefficient. It is independent of the NMR spectrometer or sample diversity with an average standard deviation of only *σ*(log *D*_x,norm_) = 0.003 in TOL-*d*_8_ and 0.002 in THF-*d*_8_ (ESI, S-Table 1 and S-Table 2‡). This experiment demonstrates the robustness of eqn (3) and the normalized diffusion coefficients.

**Table 1:** log *D*_ref,fix_ values of the used internal references

Internal reference	log *D*_ref,fix_ ^*a*^
ADAM in TOL-*d*_8_ ^*b*^	–8.8454
TMB in THF-*d*_8_ ^*c*^	–8.7749

^*a*^Each diffusion coefficient was estimated by using the middle log *D* value of 15 DOSY measurements of 15 mM solutions at 25 °C.

^*b*^ADAM has two signals in the ^1^H-NMR-spectrum. For determining the diffusion coefficient, we always used the signal of the –CH_2_ groups with the highest intensity.

^*c*^The chemical shift of one ADAM signal is very close to the THF-*d*_7_ signal at 1.73 ppm. Therefore we used TMB as internal reference for all THF-*d*_8_ solvates.

### External calibration curves and internal references

**Fig. 2:**
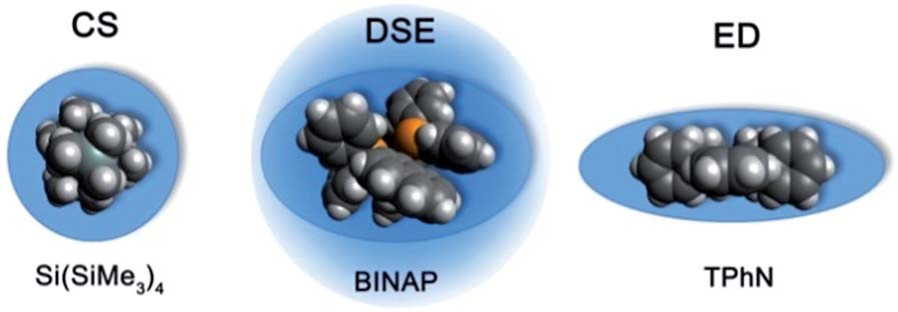
Example molecules that were classified in our calibration curves as CS, DSE and ED like molecules.

The MW-determination developed by Li and Williard *et al.* relies on an internal calibration curve (ICC) that has been generated by a single DOSY measurement where all references are present in the same NMR sample. The calibration curves which are presented in this article were generated by measuring 28 model compounds in independent NMR samples. That is the reason why we name these calibration curves “external”. The calibration curves have been plotted the common way by linearizing^22^ the power law (2) with taking the logarithm of both sides (see ESI,‡ chapter IV).^23^ Plotting log *D versus* log MW of all model molecules in one diagram gives a linear correlation but with a significant deviation especially for the very low and higher weight molecules that prevents accurate MW-determination (Fig. 1). To find a better correlation between diffusion coefficient and the MW we generated three dimensional, shape optimized models of all model compounds. By comparing the diffusion coefficient of each molecule with its shape and compactness features, one can see a significant disparity that prompted us to separate the molecules into three different types: (1) compact spheres (CS), (2) dissipated spheres and ellipsoids (DSE) and (3) expanded discs (ED) (Fig. 1).^24^ Of course the transitions between those types occur across a foggy line but there are clear systematic trends that can be rationalized. From Fig. 2 it is obvious that CS have nearly the same radius in all dimensions with a high-density space like for example the compact molecules ADAM or Si(SiMe_3_)_4_.

**Fig. 3:**
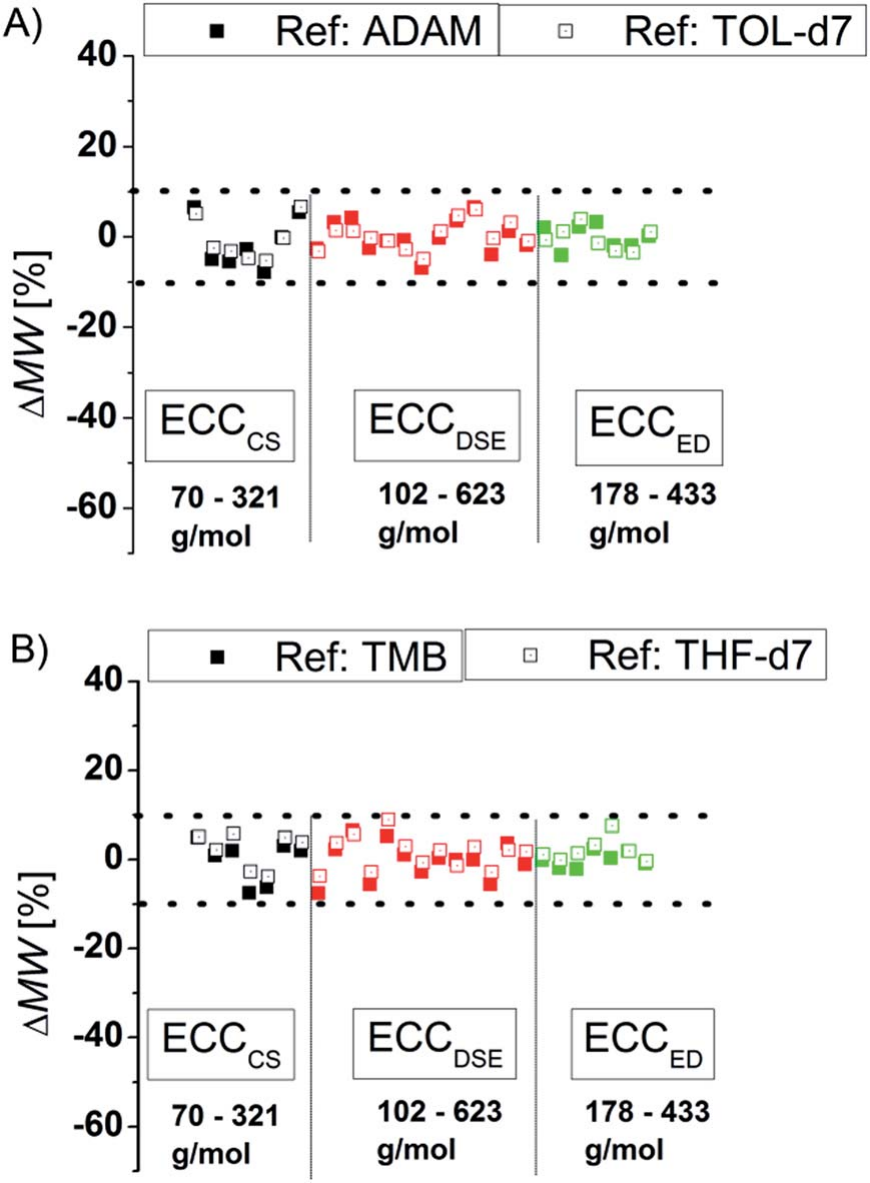
Using (A) ADAM/TOL-*d*_7_ or (B) TMB/THF-*d*_7_ as internal reference (15 mM) gives a good MW-determination with a standard deviation of *σ* = 4%. All of these model compounds were used to derive the ECCs.

However, the majority of small molecules diffuse like DSE. These are either spherical-like molecules that are less compact than CS (*e.g.* compounds with dative bonds) and/or ellipsoidal molecules like *e.g.* tetramethoxypropane or 2,2-bis(diphenylphosphino)-1,1-binaphthyl (BINAP). Small aromatic compounds like toluene, indene or naphthalene with MW < 160 g mol^–1^ diffuse also like DSE molecules. The significance of two dimensional geometries begins approximately at MW > 178 g mol^–1^. This is the region where the type of the ED begins, including molecules like anthracene (178 g mol^–1^) or tetraphenylnaphthalene (TPhN, 433 g mol^–1^). Depending on these different molecular types^25^ we plotted their log *D versus* log MW in one separate plot. These give excellent linear fits with a small error and very high *R* ^2^ values of *R* ^2^ ≈ 1 (see ESI, S-Fig. 2 and S-Fig. 3‡). Those fits result in six different ECCs: ECCSCS, ECCSDSE and ECCSED, each for every solvent (S = TOL-*d*_8_ or THF-*d*_8_). The linear fit parameters are summarized in Table 2. We also merged all calibration curves to generate a merged ECCSmerge.

**Table 2:** Linear fit parameter for the four ECCs each for TOL-*d*_8_ and THF-*d*_8_ solutions

S	TOL-*d*_8_		THF-*d*_8_	
	–log *K*	–*α*	–log *K*	–*α*
ECCSCS	7.7581	0.5018	7.7427	0.4943
ECCSDSE	7.5197	0.6098	7.5360	0.5824
ECCSED	7.1008	0.7836	7.1205	0.7519
ECCSmerge	7.4595	0.6318	7.4664	0.6095

*α* is related to the Flory exponent that comes from the fractal theory and can be described as a measure of compactness of a molecular shape. A Flory exponent of –*α* = 0.33 notes that the space is totally filled and no holes are left. On the other hand a Flory exponent of –*α* = 1 means that the molecule is completely one-dimensional and extends linearly like a rigid rod.^11^ The estimated *α*-values from the ECCs stay in very good agreement with the Flory exponent. The ECCs for compact spheres have a low *α*-value (–*α* ≈ 0.5) and the ECCs for the less compact spheres and ellipsoids have a higher *α*-value (–*α* ≈ 0.6). The two dimensional huge discs have the highest Flory exponent (–*α* ≈ 0.8) like expected.

When internal calibration curves (ICC) are used, then all references have to be in the same sample. The diffusion coefficients of those internal references show a linear dependency. In our external calibration curves (ECC), (where each model compound has been measured with ADAM or TMB as internal reference), we also see a linear behavior. These compounds behave as they were all measured in the same NMR sample. Therefore the idea occurred that beside ADAM and TMB, basically all model compounds could act as internal references for the ECCs, according to:

4 log *D*_x,norm_ = log D*ref,fix

Consequently we measured DOSYs with some model compounds (*e.g.* ADAM + Si(SiMe_3_)_4_ + naphthalene in TOL-*d*_8_) in the same NMR sample and used every compound as an internal reference by applying eqn (4). In fact it is possible to determine accurate MW of all compounds by using the normalized log *D*_x,norm_ value as fixed reference (Table 3). Utilizing *e.g.* TOL-*d*_7_ as internal reference, ADAM, Si(SiMe_3_)_4_ and naphthalene can be determined with an average deviation of ±5%. That means that the “real” molecular weight was missed by only 5%, although we used a calibration curve on the basis of many references that were not present in this NMR sample. With the ECC it is possible to simulate a bench of internal references by adding just one of them to the NMR sample. All 28 compounds behave like they were all measured in the same NMR sample. This interrelation has the colossal advantage that it is unnecessary to introduce all references into the same NMR sample. Signal overlapping, analyte–reference interaction problems, wasting chemicals and deuterated solvents can be avoided, *i.e.* saved.

**Table 3:** Mixed composition of compounds (each 15 mM) in TOL-*d*_8_ acting themselves as internal reference for the ECC^TOL^-MW-determination

Analyte	MW [g mol^–1^]	Ref 1 TOL-*d*_7_		Ref 2 ADAM		Ref 3 Si(SiMe_3_)_4_		Ref 4 naphthalene	
		MW_det_ [g mol^–1^]	ΔMW [%]	MW_det_ [g mol^–1^]	ΔMW [%]	MW_det_ [g mol^–1^]	ΔMW [%]	MW_det_ [g mol^–1^]	ΔMW [%]
TOL-*d*_7_ ^*b*^	99	96	3	97	2	96	3	97	2
ADAM^*a*^	136	144	–6	147	–8	144	–6	145	–7
Si(SiMe_3_)_4_ ^*a*^	321	304	5	309	4	303	5	305	5
Naphthalene^*b*^	128	122	5	124	3	122	5	122	5

^*a*^ECCTOLCS was used to calculate the MW.

^*b*^ECCTOLDSE was used to calculate the MW.

Due to the normalized diffusion coefficients everyone can use the ECCs, independent of the NMR device, without always recording new calibration curves. Inert compounds that are suitable to act as internal reference and their log D*ref,fix values are summarized in Table 4. In the next section the use of the residual solvent signal of TOL-*d*_8_ and THF-*d*_8_ (that arises from the proton of isotopomers containing one less deuterium atom than the perdeuterated solvent)^26^ as internal reference is examined in detail. In the following those isotopomers are referred to as TOL-*d*_7_ and THF-*d*_7_, respectively.

**Table 4:** Overview of all ECC-adapted references that fulfill the requirement of internal references for 15 mM solutions. All TOL-*d*_8_ solvates were normalized to ADAM and all THF-*d*_8_ solvates were normalized to TMB

MW [g mol^–1^]	Compound^*a*^	TOL-*d*_8_ log D*ref,fix	THF-*d*_8_ log D*ref,fix
70	Cyclopentane	–8.6694	–8.6437
79	THF-*d*_7_ ^*b*^	—	–8.6335
88	TMS	–8.7445	–8.7018
92	TOL	—	–8.6742
99	TOL-*d*_7_ ^*d*^	–8.7289	—
114	TMB	–8.7963	–8.7749^*c*^
116	Indene	–8.7698	–8.7325
128	Naphthalene	–8.7932	–8.7461
136	ADAM^*e*^	–8.8454^*c*^	—
178	Diphenylacetylene	–8.9095	–8.8535
178	Anthracene	–8.8574	–8.8129
192	9-Methylanthracene	–8.8824	–8.8440
202	Pyrene	–8.8960	–8.8457
204	1-Phenylnaphthalene	–8.9184	–8.8812
228	Triphenylene	–8.9552	–8.8869
321	Si(SiMe_3_)_4_	–9.0038	–8.9773
433	Tetraphenylnaphthalene	–9.1660	–9.1054

^*a*^When a compound had more than one ^1^H signal, the average diffusion coefficient was used.

^*b*^Due to the very high access of the solvent, the signal of THF-*d*_7_ can be used as internal reference, but a higher MW_det_ error can occur, when the solvent is coordinating to *e.g.* a metal.

^*c*^The “original” log *D*_ref,fix_ values that were used for all ECCs.

^*d*^We calculated the middle diffusion coefficients of the three aromatic protons. The final diffusion coefficient was calculated by middling this value with the diffusion coefficient of the methyl group at 1.73 ppm.

^*e*^ADAM has two signals in the ^1^H-spectrum. For determining the diffusion coefficient, we always used the signal of the –CH_2_ groups with the highest intensity.

### Quality of TOL-*d*_7_ and THF-*d*_7_ as internal reference

To compare the quality of the ECCs we calculated the MWs of all model compounds by using the log D*ref,fix values of TOL-*d*_7_ and THF-*d*_7_ as internal references. The MWs of the model compounds were determined by using the appropriate ECCs. The MW of cyclopentane (Fig. 3, very left point, ΔMW = 7%) was *e.g.* determined by using ECC_CS_ and TPhN (Fig. 3, very right point, ΔMW = 0%) by using ECC_ED_. Fig. 3 shows that the quality of MW-determination is reference-independent. It does not matter if ADAM/TMB or TOL-*d*_7_/THF-*d*_7_ were used as internal reference. Both give excellent MW-predictions with a standard deviation of 4%. The maximum error in both solvents is ±9% so we postulate that this is the maximum resolution of this DOSY method.

### Influence of the shape

**Fig. 4:**
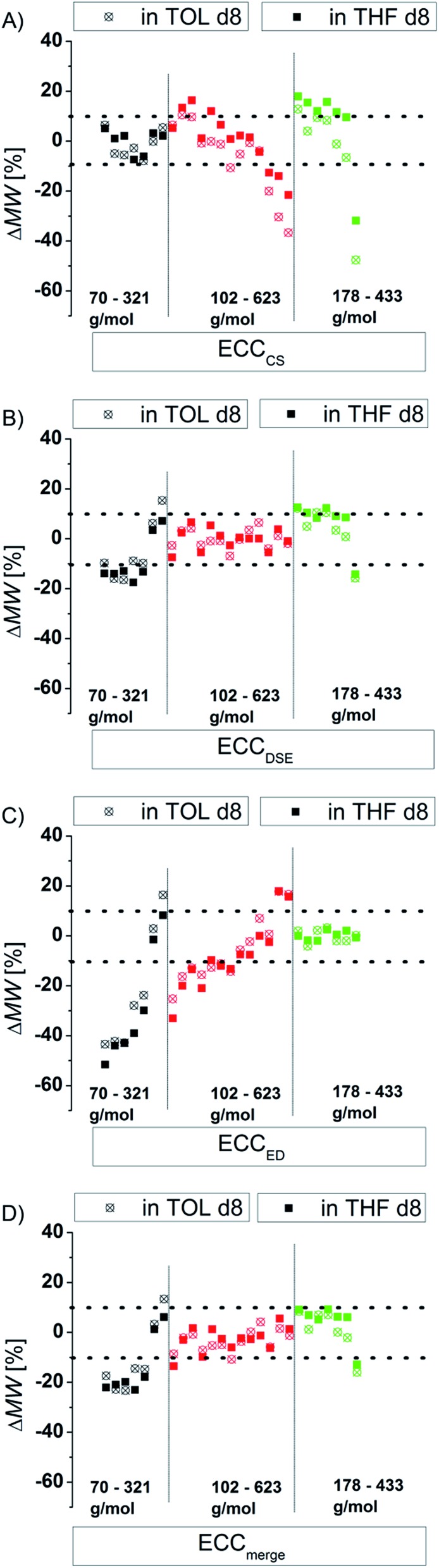
Using exclusively (A) ECCSCS, (B) ECCSDSE, (C) ECCSED and (D) ECC_merge_ on all model compounds (15 mM).

There are several modifications of the Stokes–Einstein equation which take the molecule's shape into account (by adding *e.g.* correction factors).^6,8^ The power law derived MW-determinations distinguish mostly between compound classes,^11^ large^9^ and small molecules,^15^ but not directly between different shapes within those molecular classes. In this section we will demonstrate that the accuracy of the power law derived MW-prediction is highly affected, also for small molecules, by the analyte's shape. To validate this issue we determined the MWs of all compounds using *e.g.* the ECCSED for expanded discs or ECCSDSE for ellipsoidal model molecules, *etc.* (Fig. 4). When for example the ECCSCS (that is for compact spherical molecules) is used on expanded disc like molecules, the determined MW will have a massive error especially for big molecules (Fig. 4A). Anticipating for example TPhN (an expanded flat disc, 433 g mol^–1^) in TOL-*d*_8_ to be spherical would produce a MW_det_ of 639 g mol^–1^ that is a 48% overestimated mass (Fig. 4A). Using *e.g.* the ECCSED for non-oblate molecules would produce especially for small molecules <170 g mol^–1^ a large error (Fig. 4C). The merged calibration curve ECCSmerge (Fig. 4D) determines MWs in a range of ΔMW = ±23%. But the deviation is much smaller in the region of approximately 120–200 g mol^–1^. On one hand that means that in this MW-region all molecules diffuse more or less independently from their shape. On the other hand the MW-determination (and the self-diffusion) of molecules that are outside that region, is increasingly influenced by the analyte's shape. Using the wrong calibration curve (or for example wrong molecules for an ICC) can produce highly erroneous MW-values. This is the reason why other calibration curves^8a,15,27^ that correlate a bundle of different molecules without considering the right shape, lead to bigger deviations from the correct MWs in the range of ΔMW = ±30%. By taking the correct shape into account it is possible to determine more accurate MWs with a deviation of ΔMW < ±9% (see Fig. 3).

### Influence of the concentration on TOL-*d*_8_ solvates

**Fig. 5:**
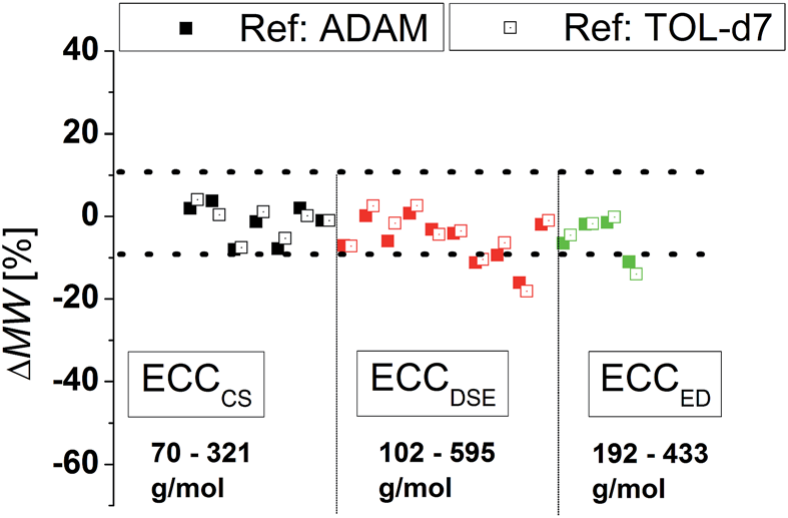
ECC-MW-determination of a few model compounds at a concentration of 120 mM at 25 °C. ADAM was used in equimolar concentration as internal reference.

All above-mentioned ECCs were derived from 15 mM solutions. We wanted to test how good the ECC-MW-determination works when the concentration is much higher than 15 mM. Therefore DOSYs of some model compounds have been measured at concentrations of 120 mM. The results are shown in Fig. 5. The Stokes–Einstein equation is only valid for infinite diluted solutions. Therefore the error should be much bigger with high concentration solutions. However, the average deviation of the estimated MW is only a little higher and most of the compounds are still in the ±9% range. The biggest error arises probably due to intermolecular interactions that result in higher estimated MWs. Especially π-interactions of big aromatic systems, at high concentrations could significantly increase the error of estimated MWs. Anyway, all compounds without aromatic rings have been determined within ±9% deviation. The power law seems to be valid even for higher concentrations, if inter- or intramolecular interactions can be excluded.^28^

### Influence of the temperature

**Fig. 6:**
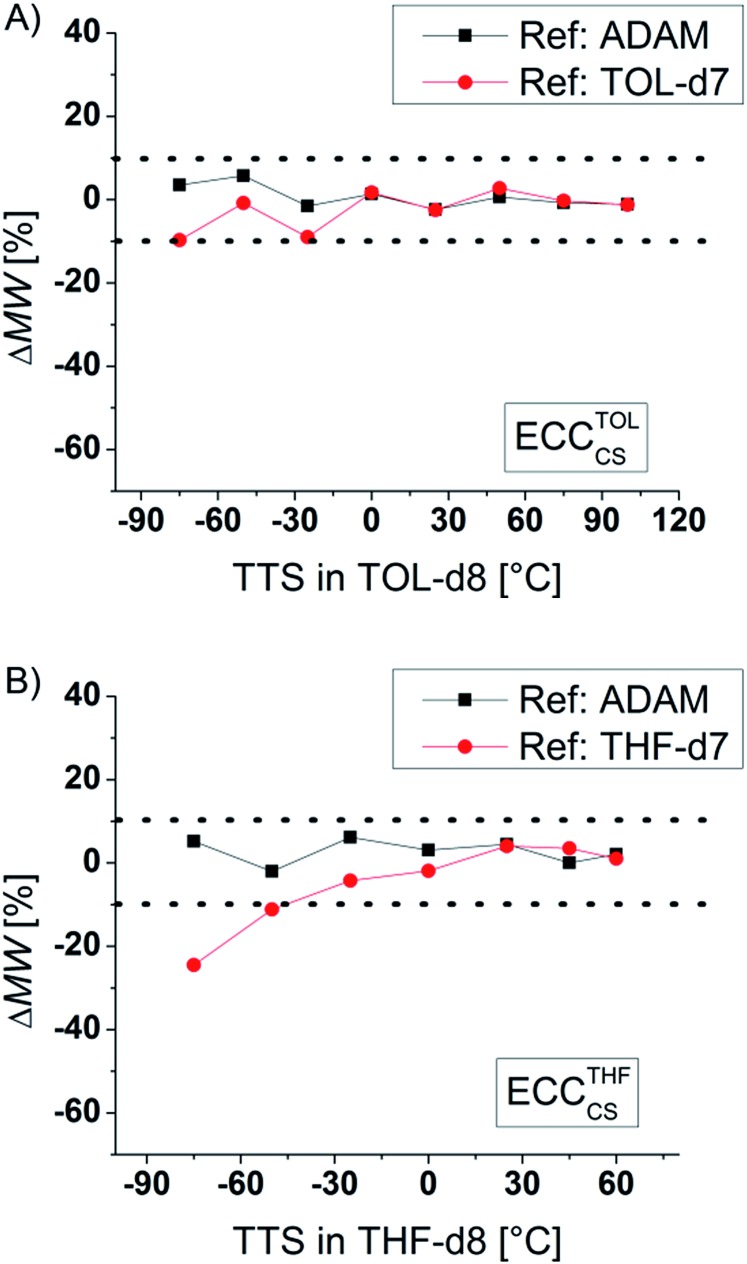
ECC-MW-determination of TTS (15 mM) in (A) TOL-*d*_8_ and in (B) THF-*d*_8_ at different temperatures.

According to the Stokes–Einstein equation the self-diffusion should be inversely proportional to the viscosity. Indeed, increasing the solvent viscosity by cooling the NMR sample from room temperature to –75 °C leads to an increase of the diffusion coefficient by almost two magnitudes! Thanks to the internal reference, the ECC-MW-determination of Si(SiMe_3_)_4_ (TTS, 321 g mol^–1^) in the full range of –75 °C to +100 °C still gives good results (Fig. 6). The internal reference is able to compensate for viscosity changes in the solvent.^23^ Notably the MW-determination is still possible at temperatures close to the boiling point of the solvent. This would give the opportunity to observe species that are forming during reactions at elevated temperatures. Furthermore, it is obvious that the signal of TOL-*d*_7_ is a useful internal reference for both high and low temperatures. But using more polar THF-*d*_7_ below –50 °C can get problematic. This probably results from solvent–solvent interactions, *i.e.* hydrogen bonding. Anyhow, it is advisable to use non-polar references and low concentrations for low temperature measurements.^28^

### Influence of halides and molecular density

In the Stokes–Einstein equation the diffusion coefficient relies on the shape and on the hydrodynamic radius of the particle. The latter can be described by the volume of the surrounding solvent molecules, the electron cloud and also the volume of the atoms. One has to keep in mind that the volume of an atom is not proportional to its atom weigh. Especially halides, compared to their atomic radii, have very high atomic masses and therefore a high mass density. For instance a potassium cation has an ion radius of 138 pm and an atom weight of 39 g mol^–1^. An iodine atom has almost the same radius of 133 pm but an atom weight of 127 g mol^–1^ that is 320% bigger than that of the K^+^ cation.^29^ Our ECCs were elaborated with references that consist mostly of hydrocarbons with some heteroatoms of the third period like silicon, phosphorus and sulfur. Therefore especially compounds containing heavy halides will be underestimated in MW. While chlorine-containing compounds are estimated with good accuracies the much denser bromides show bigger deviations from the correct MWs (see Table 5). Especially an increasing halide/carbon ratio leads to bigger errors. For example triphenylmethylbromide with one bromine atom is underestimated by 12%. But 9,10-dibromoanthracene with two bromides, a small carbon backbone and therefore a very high molar density is underestimated in MW by 42%. That means that the power law depends heavily on the molecular density.

**Table 5:** ECC-MW-determination of molecules with halides

Compound	MW [g mol^–1^]	MW_det_ ^*a*^ [g mol^–1^]	ΔMW [%]	MD_w_/10^29^ [g mol^–1^ m^–3^]
1-Hexylchloride	120	117	2	5.49
1-Octylchloride	149	143	4	5.29
1-Decylchloride	177	176	1	5.13
1-Propylbromide	123	82	34	9.66
Triphenylmethylbromide	323	283	12	6.45
9,10-Dibromoanthracene	336	194^*b*^	42	8.71
1-Butyliodide	184	102	45	11.15

^*a*^ECC_DSE_ was used to determine the MW.

^*b*^ECC_ED_ was used to determine the MW.

There are more or less extensive ways to calculate the density of a molecule. We decided to derive a simple but robust equation that correlates the MW to the approximated volume of a compound. Therefore eqn (5) was derived, where MW is the molecular weight and *V*_W_ is the van der Waals-volume of an atom. In respect to this equation we calculated the van der Waals-volumes of all atoms of a compound and summed them up to one single van der Waals-sphere (*V*_Sph_) (see ESI, S-Table 11‡).^30^ Of course this method is just an approximation without considering the real covalent bond–bond distances and the shape of the compounds. But the ratio between the MW and the sum of all van der Waals-volumes (*V*_w_) leads to a value that represents approximately a molar van der Waals-density (MD_W_) in a unit of g mol^–1^ m^–3^:

5 MD_W_ = MW/∑*V*_W_ = MW/*V*_Sph_

**Fig. 7:**
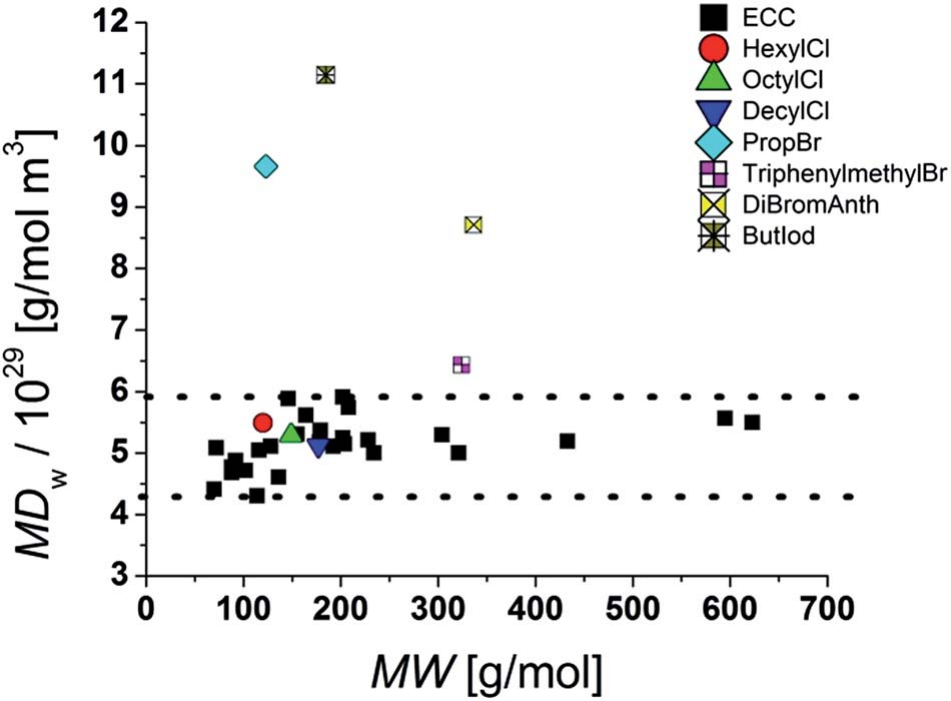
MD_W_ distribution in the model compounds and molecules with heavy atoms.

Plotting MD_W_ against MW give for our model compounds an average density distribution of around 5.2 × 10^29^ g mol^–1^ m^–3^ (Fig. 7).

Obviously the ECCs presented in this article work well with molecules with a molar density between 4.3 × 10^29^ g mol^–1^ m^–3^ and 5.9 × 10^29^ g mol^–1^ m^–3^. Higher molar van der Waals-densities like for example 9,10-dibromoanthracene [MD_W_ = 8.7 × 10^29^ g mol^–1^ m^–3^] will be underestimated and lower MD_w_ values will be overestimated in MW. To obtain accurate MWs for molecules with high densities it is necessary to measure a new calibration curve with references of comparable molar densities and geometries. Additional studies are in progress.

### Deuterated compounds

The MW-estimation of the residual solvent peak (THF-*d*_7_, 79 g mol^–1^) gives a MW of 63 g mol^–1^ that would be underestimated in MW (ΔMW = 20%). The determined MW fits much better to the protonated THF-h_8_ species (72 g mol^–1^, ΔMW = 8%). This is congruent to the nearly similar atomic radius of D compared to H. According to the above-mentioned correlation of the atomic volume and the corresponding diffusion coefficient, it is clear that deuterated molecules diffuse approximately like their protonated counterparts, although they have slightly bigger MWs. In the case of TOL-*d*_7_ (99 g mol^–1^) this effect is less pronounced (MW_det_ = 96 g mol^–1^, ΔMW = 3% rel. to TOL-*d*_7_, ΔMW = –5% rel. to TOL-h_8_) due to the relative higher MW of toluene. Moreover, especially in the case of multiple THF-*d*_8_-coordination it is advisable to use the molecular weight of THF-h_8_ to have an accurate MW-determination.

### Determining the MW of alkali organometallics

On the one hand organometallic compounds tend to aggregate *via* coordinative bonds that are significantly longer than covalent bonds. Additionally solvent molecules can associate and dissociate in solution.^31^ Therefore we anticipate that the space between all atoms is less packed than in the “sigma bonded-compact spheres” model. On the other hand alkaline organometallics frequently adopt spherical and ellipsoidal shapes, according to the ring-stacking principle.^32^ This is why we think that the ECCSDSE for “dissipated spheres and ellipsoids” is the best calibration curve for s-block organometallic compounds. As a proof of principle we made an ECCTHFDSE-MW-determination of LDA in THF-*d*_8_ solution^33^ (15 mM). THF solvated LDA is known to exist exclusively as a disolvated dimer (MW = 358 g mol^–1^).^34^ In fact, using ECCTHFDSE estimates a MW of MW_det_ = 347 g mol^–1^ with a deviation of only 3% (see Table 6).

**Table 6:** ECC-MW-determination of various lithium organics in solution. ECC_DSE_ was used to determine the MWs

Species	MW	MW_det_	ΔMW
[(Me_2_CH)_2_NLi(THF)]_2_	358	347^*a*^	3
[Me_2_NC_6_H_4_Li]_4_	508	527^*b*^	–4
[(Me_2_NC_6_H_4_Li)_3_(^*t*^BuLi)]	445	435^*b*^	2
[(Me_2_NC_6_H_4_Li)_2_(^*t*^BuLi)_2_]	382	367^*b*^	4
[(Me_2_NC_6_H_4_Li)(^*t*^BuLi)_3_]	319	316^*b*^	1
[^*t*^BuLi]_4_	256	244^*b*^	5

^*a*^1-Phenylnaphthalene was used as internal reference.

^*b*^TOL-*d*_7_ was used as internal reference.

Recently we showed that *ortho* lithium dimethylaniline (Me_2_NC_6_H_4_Li) crystallizes in the presence of *tert*-butyl lithium (^*t*^BuLi) as a separated lithium organic aggregate [^*t*^BuLi]_4_·4[Me_2_NC_6_H_4_Li]_4_ in the same crystal.^4*l*^ Dissolving those crystals in TOL-*d*_8_ resulted in an unexpectedly complicated ^7^Li NMR spectrum that shows five relatively sharp distinguishable signals over a range of nearly 2.5 ppm. These compounds where analysed by ^7^Li-DOSY experiments and were anticipated to belong to homo and heteroleptic tetramers of *o*-lithium anilide and ^*t*^BuLi. The ECCTHFDSE-MW-determination confirms those results (see Table 6).

### Determining the MW of Na-indenide in THF

**Fig. 8:**
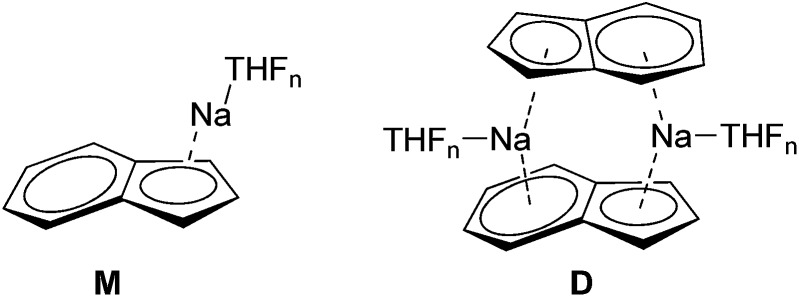
Most plausible Na-indenide species.

Alkali metal indenides are important precursors for the synthesis of metallocenes of the main group and transition metals. Without donating ligands like THF, dimethoxyethane (DME) or chelating crown ethers *etc.* they build up polymeric stack structures.^35^ The solid-state structure of base-free Na-indenide is unknown. With donating ligands Li- and Na-indenide with PMDETA or crown ethers form contact ion pairs (CIP)^36^ and with ammonia solvent separated ion pairs (SSIP).^37^ There are no investigations about the aggregation of Na-indenide in solution. One reason for that may be the relatively bad NMR properties of the sodium nucleus that has a spin of 3/2.^38^ This quadrupole results in broad lines that get even broader with asymmetry of the environment. The ^1^H-DOSY experiment is independent of that nucleus. Therefore Na-indenide is an interesting candidate for discovering its aggregation in solution. The most feasible species are the THF solvated monomers (**M1–M4**) and the dimers **D1–D2** (Fig. 8). The molar density for all species is between MD = 5.07 and 5.43 × 10^29^ g mol^–1^ m^–3^ which ensures that those aggregates are suitable for our calibration curves (see Fig. 7).

At room temperature (RT) the ECCTHFDSE-MW-determination estimates a MW of MW_det_ = 331 g mol^–1^. The comparison of that MW with the most likely Na-indenide species (**1–7**) is shown in Table 7. Both dimers with two- (**D1**: ΔMW = 21%) and four THF molecules (**D2**: ΔMW = 41%) can be excluded. The same is true for the mono- (**M1**: ΔMW = –57%) and disolvated monomers (**M2**: ΔMW = –17%). The tri-solvated monomer (**M3**: ΔMW = 7%) gives the best match. Such a three-fold THF coordination fits perfectly many crystal structures of THF solvated sodium cyclopentadienide derivatives.^39^ We can also identify without difficulty the signals of remaining indene (ΔMW = 2%) and hexamethyldisilazane ((H)HMDS, ΔMW = –5%). Those very accurate MWs indicate that the exchange of the latter with Na-indenide **M3** is very slow or not present. Otherwise the estimated MWs of indene or (H)HMDS should be much higher.

**Table 7:** ECC-MW-determination of Na-indenide (15 mM) in THF-*d*_8_ at various temperatures. TMB (15 mM) was used as internal reference and ECCTHFDSE to determine the MWs

Species	*n*	MW [g mol^–1^]	ΔMW [%]		
			–50 °C	+25 °C	+60 °C
**M1**	1	210	–84	–57	–36
**M2**	2	282	–37	–17	–1
**M3**	3	354	–9	7	19
**M4**	4	426	10	22	33
**D1**	1	420	8^*a*^	21	32
**D2**	2	564	32	41	49
Indene		111	4	2	–30
(H)HMDS		161	–5	–5	–1

^*a*^The disolvated dimer **D1** (ΔMW = 8%) would also fit to the estimated MW, but this aggregation makes in this context chemically not much sense.

At –50 °C it is obvious that the equilibrium of Na-indenide changes to a higher MW of MW_det_ = 386 g mol^–1^. That MW is right in between three- (**M3**: ΔMW = –9%) and four-fold (**M4**: ΔMW = 10%) THF-coordinated Na-indenide, indicating that a fourth THF coordination is attractive at low temperatures. Again, indene (ΔMW = 4%) and (H)HMDS (ΔMW = –5%) are not involved in that Na-indenide–THF equilibrium. By warming up the THF solution to +60 °C the opposite trend is evident. The ECC-MW-determination estimates for Na-indenide a much lower MW of MW_det_ = 286 g mol^–1^ that would fit to a disolvated Na-indenide monomer (**M2**: ΔMW = –1%) but additionally the MW of indene rises significantly to (MW_det_ = 158 g mol^–1^, ΔMW = –37%). That indicates that there is a rapid exchange of Na-indenide and indene at high temperatures producing a merged MW for both. Anyway, HMDS is still not involved in that equilibrium (ΔMW = –1%) perhaps due to its higher basicity and steric demand, compared with indene (p*K*_s_ = 26 *vs.* 20).^40^

## Conclusions

In this article we described how to determine very accurately and reliably molecular weights from DOSY measurements. We derived the equation (log *D*_x,norm_ = log *D*_ref,fix_ – log *D*_ref_ + log *D*_x_) that facilitates defining fixed and normalized diffusion values. This approach enables the use of only one internal reference to determine accurate molecular weights within a deviation of only ΔMW < ±9%. We showed that the ECC-MW-determination is valid at a wide temperature range, enabling to monitor reactions and intermediates at low and high temperatures. Further we showed how the shape and the molar density of compounds influence the power law derived MW-determination. We derived an equation that calculates a term of a molar van der Waals-density that helps to approximate, whether molecules are valid for specified calibration curves or not. In the end we used the ECC-approach to characterize the aggregations of Na-indenide at various temperatures. The ECC-method could easily be extended to other compounds, complexes or solvents giving the opportunity to develop a database of external calibration curves and internal references that would enable access to accurate MW-determination for everyone. An Excel spreadsheet that allows calculating MWs from diffusion coefficients is available for download from the authors' web site (see ESI‡).

## Experimental

TOL-*d*_8_ and THF-*d*_8_ (99.8%, Aldrich, with little amounts of water) were used for the calibration curves. In the case of the Na-indenide, dry THF-*d*_8_ was used that was kept with 4 Å molecular sieves under argon. All samples were prepared by adding adamantane (ADAM, in TOL-*d*_8_) respectively 2,2,3,3-tetramethylbutane (TMB, in THF-*d*_8_) and analyte (each 15 mM, when not mentioned otherwise) in an equimolar ratio. The diffusion coefficients of the model compounds in TOL-*d*_8_ were normalized to the ADAM signal with a fixed value of log *D*_ref,fix_(ADAM) = –8.8454. THF solvates were normalized to the TMB signal to the fixed value of log *D*_ref,fix_(TMB) = –8.7749. NMR experiments were recorded on two devices: (1) Bruker Avance 400 spectrometer equipped with an observe broadband probe with *z*-axis gradient coil with maximum gradient strength of 57 G cm^–1^ and (2) Bruker Ascend 400 spectrometer equipped with an inverse broadband probe with *z*-axis gradient coil with maximum gradient strength of 51 G cm^–1^. All spectra were acquired in 5 mm NMR tubes. Sample spinning was deactivated during the measurements. All DOSY experiments were performed using a double stimulated echo sequence with bipolar gradient pulses and three spoil gradients with convection compensation (dstebpgp3s).^41^ The diffusion time was *Δ* = 0.1 s. The duration of the magnetic field pulse gradients *δ*/2 was adjusted for each temperature in a range of 400–3500 μs. The delay for gradient recovery was 0.2 ms and the eddy current delay 5 ms. For each DOSY-NMR experiment, a series of 16 spectra on 32 K data points were collected. The pulse gradients (*g*) were incremented from 2 to 98% of the maximum gradient strength in a linear ramp with a total experiment time of 45 min. The temperature was set and controlled at 298 K with an air flow of 400 l h^–1^ in order to avoid any temperature fluctuations due to sample heating during the magnetic field pulse gradients. After Fourier transformation and baseline correction, the diffusion dimension was processed with the Topspin 3.1 software. Diffusion coefficients, processed with a line broadening of 2 Hz, were calculated by Gaussian fits with the T1/T2 software of Topspin.
