# Supplementary material for: Accurate molecular weight determination of small molecules via DOSY-NMR by using external calibration curves with normalized diffusion coefficients
Source: Chem Sci. 2015 Mar 19;6(6):3354–64. doi: 10.1039/c5sc00670h (PMC5656982; doi:10.1039/c5sc00670h)
Supplement: Supplementary file 1 [file SC-006-C5SC00670H-s001.pdf]

Electronic Supplementary Material (ESI) for Chemical Science.
This journal is © The Royal Society of Chemistry 2015

SUPPORTING INFO

Accurate Molecular Weight Determination of Small Molecules via DOSY-NMR by using External Calibration Curves with Normalized Diffusion Coefficients

*Roman Neufeld and Dietmar Stalke**

Institut für Anorganische Chemie,
Georg-August-Universität,
Tammannstrasse 4,
D-37077, Göttingen, Germany.

E-mail: dstalke@chemie.uni-goettingen.de

Contents

I. Definition of Δ*MW* in ECC-MW-Determination 2
II. Estimating the Maximum Error of log$D_{x,norm}$ in TOL-d_8 and THF-d_8 2
III. Overview of the Used Model Compounds for ECCs 3
IV. Creating Calibration Curves 7
V. Influence of High Concentration 11
VI. Using Various Molecules as Internal Reference within the same NMR-Sample 12
VII. Testing the Influence of the Temperature on ECCs 12
VIII. Gaussian fits of the T1/T2 software of Topspin for LDA in THF-d_8 at 25°C 13
IX. Gaussian fits of the T1/T2 software of Topspin for the $[^tBuLi]_4 \cdot 4[Me_2NC_6H_4Li]_4$ crystal in TOL-d_8 at 25°C 14
X. Gaussian fits of the T1/T2 software of Topspin for Na-indenide in THF-d_8 at 25°C 16
XI. Gaussian fits of the T1/T2 software of Topspin for Na-indenide in THF-d_8 at -50°C 17
XII. Gaussian fits of the T1/T2 software of Topspin for Na-indenide in THF-d_8 at 60°C 19
XIII. Calculation of the Molar Van-der-Vaals Density MD_w 21
XIV. ECC-MW-Determination Excel Spreadsheet 22

I. Definition of Δ*MW* in ECC-MW-Determination

To estimate how good the MW-determination works (using for example the solvent or another molecule as internal reference) we calculate the deviation of the determined MW from the "real" MW of the compound in %, according to **equation (S1)**:

$$\textbf{(S1)}\quad \Delta MW = \left[1 - {}^{MW_{det}}/_{MW}\right] \cdot 100\,\%$$

When the ΔMW is positive then the MW was determined too low and when ΔMW is negative then the MW was estimated too high.

II. Estimating the Maximum Error of log$D_{x,norm}$ in TOL-d_8 and THF-d_8

All measurements were performed at 25°C. All compounds have been measured in 15 mM solutions of analyte and reference in an equimolar ratio. The absolute diffusion coefficients (D_x) of all compounds are different on each NMR device. But the normalized diffusion coefficients log$D_{x,norm}$ shows on all devices nearly the same value with a small average standard deviation of σ = 0.0028 in TOL-d_8 and σ = 0.0020 in THF-d_8, see **S-Table 1** and **S-Table 2**.

S-Table 1: Diffusion parameter measured on two different NMR-devices (in TOL-d_8 with ADAM as internal reference).

Device 1 [a)] Compound	logD_x	log$D_{x,norm}$	logD_{ref}	***Device 2*** [b)] logD_x	log$D_{x,norm}$	logD_{ref}	**Average** log$D_{x,norm}$	**std. dev** σ
TMS	-8.6449	-8.7453	-8.7450	-8.7445	-8.7437	-8.8462	-8.7445	0.0012
ADAM	-8.7450	-8.8454	-8.7450	-8.8462	-8.8454	-8.8462	-8.8454	0.0000
$N(SiMe_3)_3$	-8.8570	-8.9525	-8.7498	-8.9454	-8.9421	-8.8486	-8.9473	0.0074
$Si(SiMe_3)_4$	-8.9090	-9.0077	-8.7467	-9.0031	-8.9998	-8.8486	-9.0038	0.0056
Cyclopentane	-8.5712	-8.6702	-8.7464	-8.6664	-8.6686	-8.8431	-8.6694	0.0011
THF	-8.5753	-8.6752	-8.7455	-8.6743	-8.6698	-8.8499	-8.6725	0.0038
TOL-d_7	-8.6334	-8.7338	-8.7450	-8.7366	-8.7358	-8.8462	-8.7348	0.0014
Indene	-8.6733	-8.7705	-8.7481	-8.7742	-8.7691	-8.8505	-8.7698	0.0010
Naphthaline	-8.7018	-8.7966	-8.7506	-8.7962	-8.7898	-8.8517	-8.7932	0.0048
2-Phenylpyridine	-8.7625	-8.8592	-8.7486	-8.8598	-8.8559	-8.8492	-8.8576	0.0023
1-Phenylnaphthaline	-8.8239	-8.9182	-8.7510	-8.9255	-8.9186	-8.8523	-8.9184	0.0003
Tri(o-tolyl)-phosphine	-8.9562	-9.0476	-8.7540	-9.0499	-9.0402	-8.8551	-9.0439	0.0053
BINAP	-9.1258	-9.2235	-8.7476	-9.2339	-9.2337	-8.8456	-9.2286	0.0072
Anthracene	-8.7630	-8.8580	-8.7503	-8.8580	-8.8569	-8.8465	-8.8574	0.0008
9-Methylanthracene	-8.7820	-8.8790	-8.7484	-8.8900	-8.8858	-8.8496	-8.8824	0.0048
Pyrene	-8.7972	-8.8937	-8.7488	-8.9030	-8.8982	-8.8502	-8.8960	0.0031
Triphenylene	-8.8593	-8.9556	-8.7491	-8.9590	-8.9548	-8.8496	-8.9552	0.0006
TPhN	-9.0772	-9.1656	-8.7570	-9.1778	-9.1664	-8.8567	-9.1660	0.0006
							**Average**	**0.0028**

a) Uncalibrated gradients
b) Calibrated gradients

S-Table 2: Diffusion parameter measured on two different NMR-devices (in THF-d_8 and TMB as internal standard).

Device 1 [a)] Compound	logD_x	log$D_{x,norm}$	logD_{ref}	***Device 2*** [b)] logD_x	log$D_{x,norm}$	logD_{ref}	Average log$D_{x,norm}$	std. dev σ
TMS	-8.5969	-8.6993	-8.6724	-8.7018	-8.7043	-8.7724	-8.7018	0.0035
TMB	-8.6724	-8.7749	-8.6724	-8.7724	-8.7749	-8.7724	-8.7749	0.0000
$N(SiMe_3)_3$	-8.8091	-8.9124	-8.6716	-8.8993	-8.9018	-8.7724	-8.9071	0.0075
$Si(SiMe_3)_4$	-8.8765	-8.9787	-8.6726	-8.9767	-8.9759	-8.7757	-8.9773	0.0020
Cyclopentane	-8.5381	-8.6439	-8.6690	-8.6428	-8.6435	-8.7742	-8.6437	0.0003
THF-d_7	-8.5303	-8.6328	-8.6724	-8.6368	-8.6393	-8.7724	-8.6360	0.0046
Indene	-8.6276	-8.7326	-8.6698	-8.7306	-8.7323	-8.7731	-8.7325	0.0002
Naphthaline	-8.6432	-8.7458	-8.6722	-8.7459	-8.7464	-8.7744	-8.7461	0.0004
2-Phenylpyridine	-8.6950	-8.7996	-8.6702	-8.7968	-8.7980	-8.7737	-8.7988	0.0011
1Phenylnaphthalin	-8.7762	-8.8799	-8.6712	-8.8918	-8.8812	-8.7854	-8.8806	0.0009
Tri(o-tolyl)-phosphine	-8.8904	-8.9914	-8.6739	-8.9965	-8.9957	-8.7757	-8.9935	0.0030
BINAP	-9.0678	-9.1666	-8.6761	-9.1670	-9.1661	-8.7757	-9.1663	0.0003
Anthracene	-8.7083	-8.8140	-8.6692	-8.8118	-8.8117	-8.7749	-8.8129	0.0016
Pyrene	-8.7453	-8.8436	-8.6765	-8.8503	-8.8479	-8.7773	-8.8457	0.0030
Triphenylene	-8.7904	-8.8889	-8.6763	-8.8852	-8.8849	-8.7752	-8.8869	0.0028
TPhN	-9.0082	-9.1057	-8.6774	-9.1103	-9.1050	-8.7802	-9.1054	0.0005
							**Average**	**0.0020**

a) Uncalibrated gradients
b) Calibrated gradients

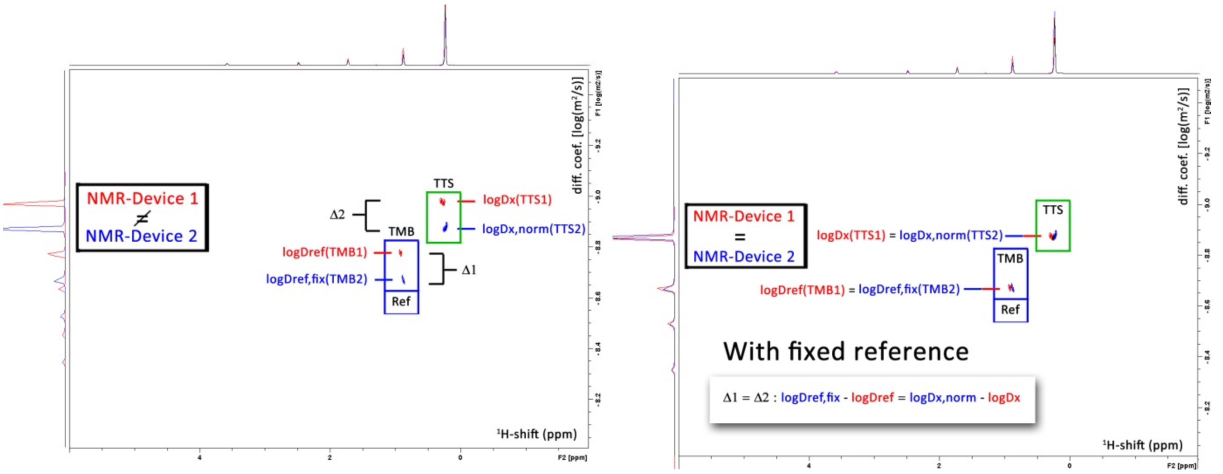

S-Figure 1: Superposition plot of two DOSY spectra measured on two different NMR devices. Left: The absolute diffusion coefficients of $Si(SiMe_3)_4$ (TTS) are uneven logD(TTS1)≠logD(TTS2) and logD(TMB1)≠logD(TMB2) due to different gradient calibrations in the NMR devices and for example diversity in viscosity and/or temperature. Right: The signal of the references has been shifted to a fixed value and the signals of TTS have been moved by the same increment of Δ1 = Δ2. With that referencing method it is possible to obtain the same diffusion values for analyte x independent of the used NMR device or changes in solution properties.

III. Overview of the Used Model Compounds for ECCs

Three dimensional models that were geometry optimized with the program Avogadro 1.1.0 have been generated. Of course the transitions between the geometries are not sharp but there are clear systematic trends that can be rationalized. **S-Table 3** one can see, that compact spherical (CS) molecules have nearly the same radius in all dimensions with a highly filled space. Dissipated spheres and ellipsoids (DSE) have an elongated main-axis and a less filled space. Small annelated aromatic compounds like toluene (92 g/mol), indene (116 g/mol) or naphthaline (128 g/mol) with MW < 150 g/mol diffuse DSE-like. Also diphenylacetylene (178 g/mol) that has an elongated molecule is still in the range of a DSE geometry. The significance of one and two dimensional

geometries begins approximately at MW > 178 g/mol. This is why the ECC_{ED} for extended discs (ED) begins with anthracene that has a MW of 178 g/mol.

S-Table 3: Classification of all model compounds appropriate to their geometries.

**MW [g/mol]**	**Compact Spheres [g/mol]**	**Dissipated Spheres and Ellipsoids [g/mol]**	**Expanded Discs [g/mol]**
**70**	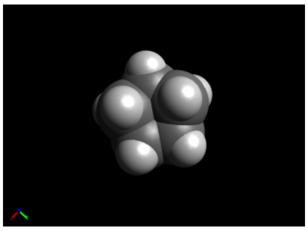 Cyclopentane (70)		
	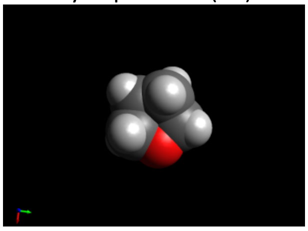 THF (72)		
	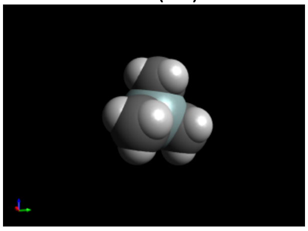 TMS (88)		
	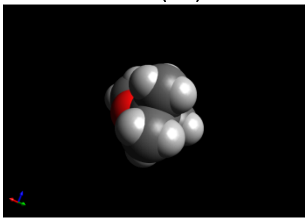 MTBE (88)		
**100**		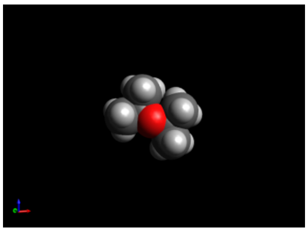 Diisopropylether (102)	
	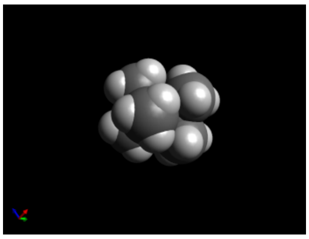 TMB (114)	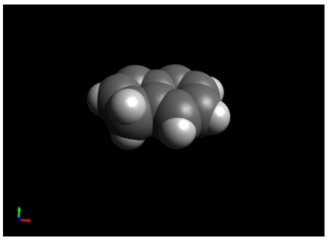 Indene (116)	

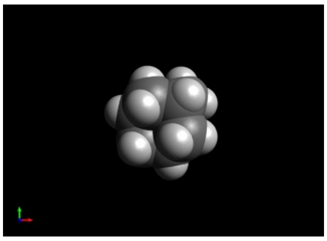

ADAM (136)

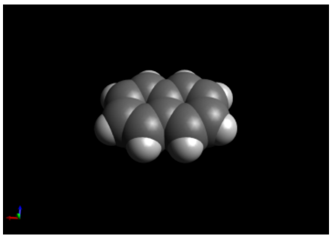

Naphthaline (128)

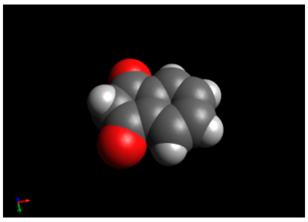

1,3-Indandione (146)

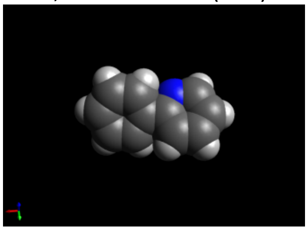

2-Phenylpyridine (155)

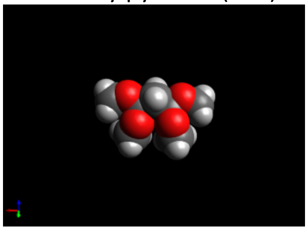

Tetramethoxypropane (164)

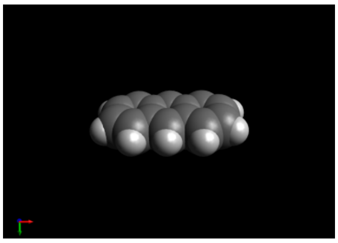

Anthracene (178)

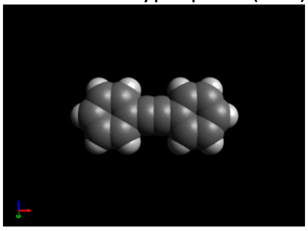

Diphenylacetylene (178)

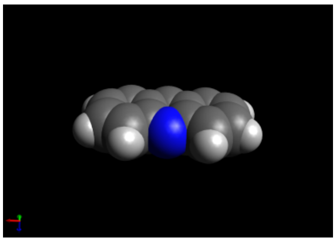

Acridine (179)

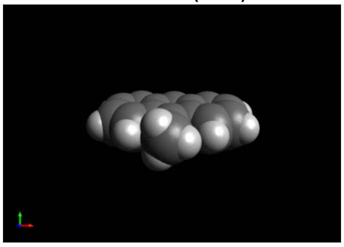

9-Methylanthracene (192)

200

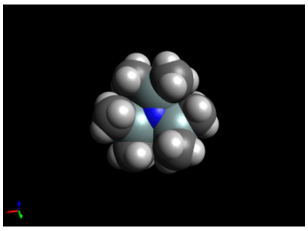

$N(SiMe_3)_3$ (234)

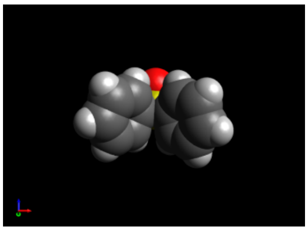

Diphenylsulfoxide (202)

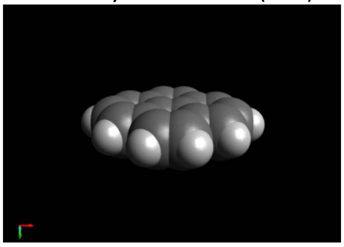

Pyrene (202)

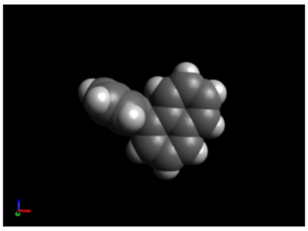
1-Phenylnaphthaline (204)

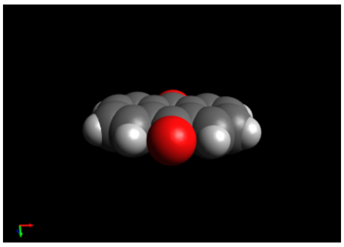
Anthtachinone (208)

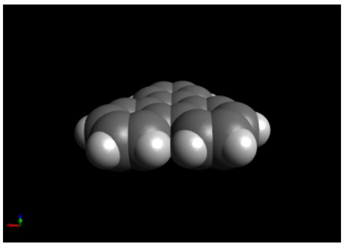
Triphenylene (228)

300

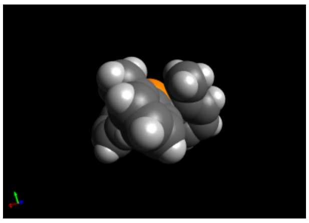
Tri-(o-tolyl)phosphine (304)

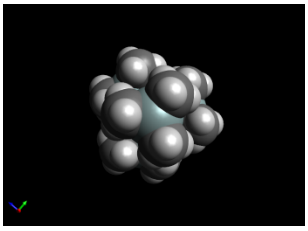
$S(SiMe_3)_4$ (321)

400

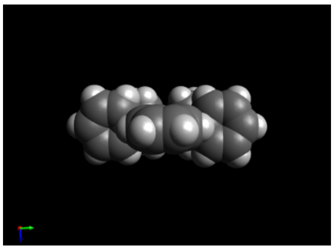
Tetraphenylnaphthaline (433)

500

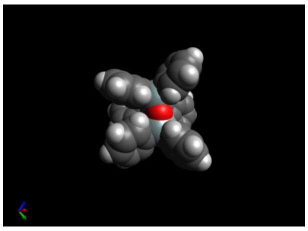
Hexaphenyltrisiloxane (595)

600

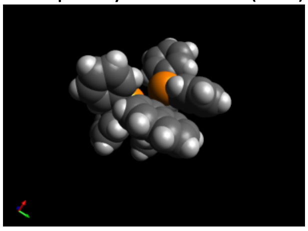
BINAP (623)

IV. Creating Calibration Curves

The power law can be linearized by taking the logarithm of both sides

$$\textbf{(S2)}\ logD = logK + \alpha\ logMW$$

$$\textbf{(S3)}\ logD_{x,norm} = logK + \alpha\ logMW$$

$$\textbf{(S4)}\ MW_{det} = 10^{\left(\frac{logD_{x,norm} - logK}{\alpha}\right)}$$

To obtain a linear correlation of *D* and *MW* we measured the diffusion coefficients of 28 different model compounds, aliphatics and aromatics with known MWs in a range of 70 $gmol^{-1}$ (cyclopentan) to 623 $gmol^{-1}$ (BINAP: (2,2'-bis(diphenylphosphino)-1,1'-binaphthyl). In TOL-d_8 we used (ADAM) and in THF-d_8 solutions we used TMB as internal standard. Plotting $logD_{x,norm}$ against $logMW$ gives a linear fit that provides the values for $logK$ and α. It is possible to calculate the MW of unknown compounds by applying their normalized diffusion coefficient $logD_{x,norm}$ to **equation (S4)**.

The maximum deviation of $logD_{x,norm}$ was 0.0075, which is approximately the width at half maximum of a DOSY signal. This is the reason why the maximum $\Delta logD_{x,norm}$ was defined as 2 times 0.0075 which is reflected in the error bars in the calibration plots:

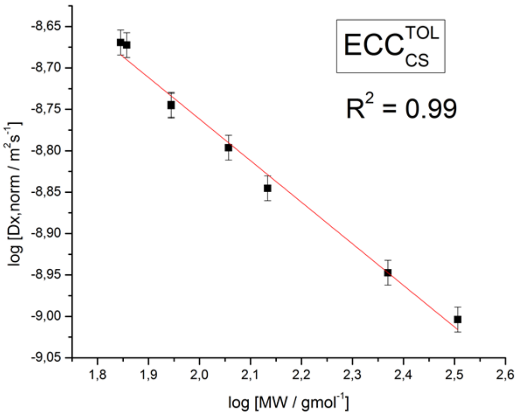

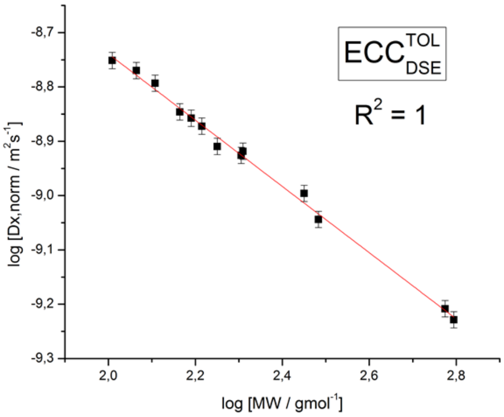

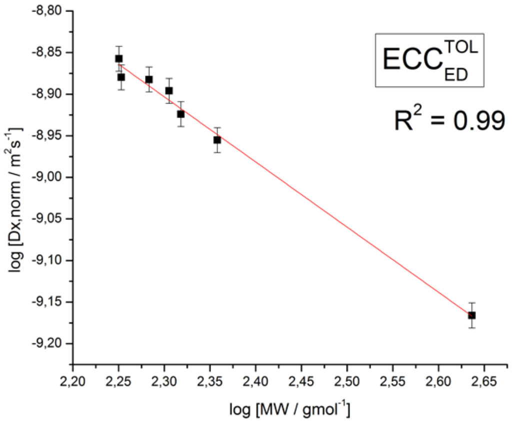

S-Table 4: ECC-parameter for TOL-d_8 solvates.

	log*K*	error	*α*	error
ECC_{CS}^{Tol}	-7.7581	0.0469	-0.5018	0.0224
ECC_{DSE}^{Tol}	-7.5197	0.0279	-0.6098	0.0120
ECC_{ED}^{Tol}	-7.1008	0.0717	-0.7836	0.0306

S-Figure 2: Plots of log$D_{x,norm}$ vs log*MW* in TOL-d_8 of all model compounds sorted by their molecular geometry. The linear fits show very high accuracy indicated by R ≥ 0.99.

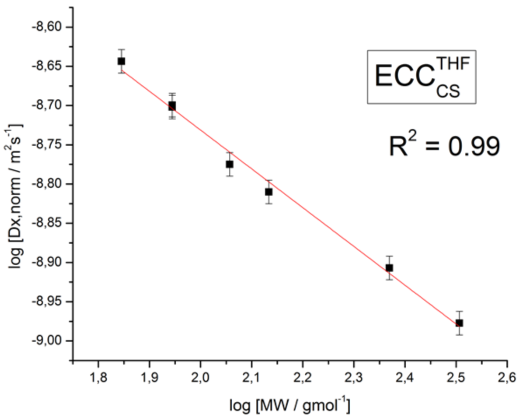

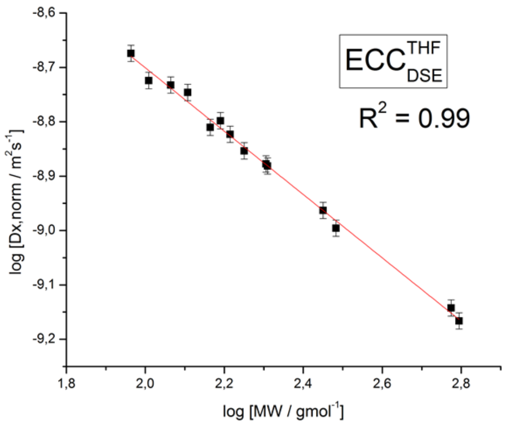

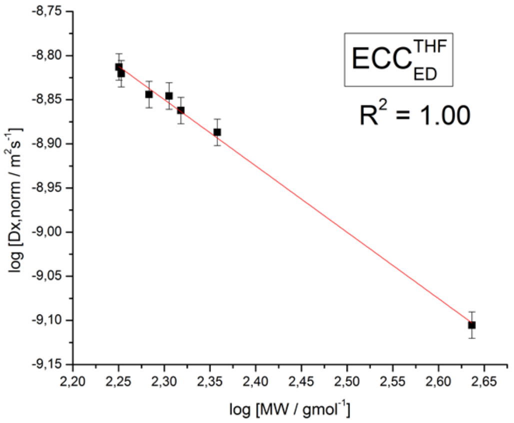

S-Table 5: ECC-parameter for THF-d_8 solvates.

	log*K*	error	*α*	error
ECC_{CS}^{THF}	-7.7427	0.0397	-0.4943	0.0187
ECC_{DSE}^{THF}	-7.5360	0.0270	-0.5824	0.0117
ECC_{ED}^{THF}	-7.1205	0.0449	-0.7519	0.0191

S-Figure 3: Plots of log$D_{x,norm}$ vs log*MW* in THF-d_8 of all model compounds sorted by their molecular geometry. The linear fits show very high accuracy indicated by R ≥ 0.99.

S-Table 6: Overview of the used model compounds for ECC^{TOL} and their normalized diffusion coefficients $\log D_{x,norm}$, the determined MW_{det} and the deviation from the real molecular weight ΔMW.[a)] ADAM was used as the internal reference with $\log D_{ref,fix}$ (ADAM) = -8.8454. All compounds have been measured in 15 mM solutions of analyte and ADAM in an equimolar ratio.

**MW [g/mol]**		**$D_{x,norm}$ [m/s²]**	**Log $D_{x,norm}$**	**Log MW_{det}**	**MW_{det} [g/mol]**	**ΔMW [%]**
	Compact Spheres, ECC_{CS}					
70	Cyclopentane	2.1411E-09	-8.6694	1.8157	65	7
72	THF	2.1258E-09	-8.6725	1.8220	66	8
88	TMS	1.8010E-09	-8.7445	1.9655	92	-5
88	MTBE	1.7965E-09	-8.7456	1.9676	93	-5
114	TMB	1.5985E-09	-8.7963	2.0687	117	-3
136	ADAM [b)]	1.4277E-09	-8.8454	2.1665	147	-8
234	$N(SiMe_3)_3$	1.1290E-09	-8.9473	2.3696	234	0
321	$Si(SiMe_3)_4$	9.9135E-10	-9.0038	2.4821	303	5
	Dissipated Spheres & Ellipsoids, ECC_{DSE}					
102	Diisopropylether	1.7727E-09	-8.7514	2.0199	105	-3
116	Indene	1.6990E-09	-8.7698	2.0501	112	3
128	Naphthaline	1.6099E-09	-8.7932	2.0885	123	4
146	1,3 Indandione	1.4257E-09	-8.8460	2.1750	150	-2
155	2-Phenylpyridine	1.3882E-09	-8.8576	2.1941	156	-1
164	Tetramethoxypropane	1.3420E-09	-8.8722	2.2181	165	-1
178	Diphenylacetylene	1.2316E-09	-8.9095	2.2793	190	-7
202	Diphenylsulfoxid	1.1859E-09	-8.9260	2.3062	202	0
204	1-Phenylnaphthaline	1.2067E-09	-8.9184	2.2938	197	4
304	Tri(o-tolyl)-phosphine	9.0389E-10	-9.0439	2.4996	316	-4
595	Hexaphenyltrisiloxane	6.1914E-10	-9.2082	2.7691	588	1
623	BINAP	5.9079E-10	-9.2286	2.8025	635	-2
	Expanded Discs, ECC_{ED}					
178	Anthracene	1.3885E-09	2.2504	-8.8574	2.2417	2
179	Acridine	1.3190E-09	2.2529	-8.8797	2.2701	-4
192	9-Methylanthracene	1.3110E-09	2.2833	-8.8824	2.2735	2
202	Pyrene	1.2707E-09	2.3054	-8.8960	2.2908	3
208	Anthrachinone	1.1915E-09	2.3181	-8.9239	2.3265	-2
228	Triphenylene	1.1087E-09	2.3579	-8.9552	2.3664	-2
433	Tetraphenylnaphthaline	6.8229E-10	2.6365	-9.1660	2.6355	0
				**Std. dev.**	**σ**	**4**

a) When a compound had more than one signal in the ^1H-NMR, the average diffusion coefficient was used.

b) For determining the diffusion coefficient, we used the signal of the $-CH_2$ groups with the highest intensity.

S-Table 7: Overview of the used model compounds for ECC^{THF} and their normalized diffusion coefficients $logD_{x,norm}$, the determined MW_{det} and the deviation ΔMW. TMB was used as the internal reference with $logD_{ref,fix}$ (TMB) = -8.7749. All compounds have been measured in 15 mM solutions of analyte and TMB in an equimolar ratio.[a)]

MW [$gmol^{-1}$]		$D_{x,norm}$ [m/s²]	Log $D_{x,norm}$	Log MW_{det}	MW_{det} [g/mol]	ΔMW [%]
	Compact Spheres, ECC_{CS}					
70	Cyclopentane	2.2713E-09	-8.6437	1.8229	67	5
88	TMS	1.9870E-09	-8.7018	1.9404	87	1
88	MTBE	1.9980E-09	-8.6994	1.9356	86	2
114	TMB	1.6793E-09	-8.7749	2.0882	123	-7
136	ADAM [b)]	1.5481E-09	-8.8102	2.1597	144	-6
234	$N(SiMe_3)_3$	1.2386E-09	-8.9071	2.3557	227	3
321	$Si(SiMe_3)_4$	1.0537E-09	-8.9773	2.4977	315	2
	Dissipated Spheres & Ellipsoids, ECC_{DSE}					
92	Toluol	2.1175E-09	-8.6742	1.9543	90	2
102	Diisopropylether	1.8871E-09	-8.7242	2.0402	110	-8
116	Indene	1.8515E-09	-8.7325	2.0544	113	2
128	Naphthaline	1.7943E-09	-8.7461	2.0778	120	7
146	1,3 Indandione	1.5478E-09	-8.8103	2.1880	154	-6
155	2-Phenylpyridine	1.5921E-09	-8.7980	2.1670	147	5
164	Tetramethoxypropane	1.5028E-09	-8.8231	2.2100	162	1
178	Diphenylacetylene	1.4013E-09	-8.8535	2.2622	183	-3
202	Diphenylsulfoxid	1.3256E-09	-8.8776	2.3035	201	0
204	1-Phenylnaphthaline	1.3146E-09	-8.8812	2.3098	204	0
304	Tri(o-tolyl)-phosphine	1.0100E-09	-8.9957	2.5063	321	-6
595	Hexaphenyltrisiloxane	7.2042E-10	-9.1424	2.7583	573	4
623	BINAP	6.8215E-10	-9.1661	2.7990	629	-1
	Expanded Discs, ECC_{ED}					
178	Anthracene	1.5386E-09	-8.8129	2.2509	178	0
179	Acridine	1.5119E-09	-8.8205	2.2610	182	-2
192	9-Methylanthracene	1.4321E-09	-8.8440	2.2923	196	-2
202	Pyrene	1.4265E-09	-8.8457	2.2946	197	2
208	Anthrachinone	1.3734E-09	-8.8622	2.3165	207	0
228	Triphenylene	1.2975E-09	-8.8869	2.3493	224	2
433	Tetraphenylnaphthaline	7.8459E-10	-9.1054	2.6399	436	-1
				**Std. dev.**	**σ**	**4**

a) When a compound had more than one signal in the ^{1}H-NMR, the average diffusion coefficient was used.
b) For determining the diffusion coefficient, we used the signal of the $-CH_2$ groups with the highest intensity.

V. Influence of High Concentration

S-Table 8: $ECC^{TOL\text{-}d8}$ from 15mM TOL-*d*8 solutions were used to determine the MW of compounds that were measured in concentrated TOL-*d*8 solutions (120 mM). The deviation ΔMW is a little bit higher than in the dilute solutions but still in a good range. ADAM was used as the internal reference with $\log D_{ref,fix}$ (ADAM) = -8.8454. [a)]

MW [$gmol^{-1}$]		$D_{x,norm}$ [m/s^2]	Log $D_{x,norm}$	Log MW_{det}	MW_{det} [g/mol]	ΔMW [%]
	Compact Spheres, ECC_{CS}					
70	cyclopentane	2.0900E-09	-8.6799	1.8367	69	2
72	THF	2.0805E-09	-8.6818	1.8406	69	4
88	MTBE	1.7751E-09	-8.7508	1.9780	95	-8
88	TMS	1.8331E-09	-8.7368	1.9502	89	-1
136	ADAM [b)]	1.4277E-09	-8.8454	2.1665	147	-8
234	$N(SiMe_3)_3$	1.1410E-09	-8.9427	2.3605	229	2
321	$Si(SiMe_3)_4$	9.5908E-10	-9.0181	2.5108	324	-1
	Dissipated Spheres & Ellipsoids, ECC_{DSE}					
102	Diisopropylether	1.7278E-09	-8.7625	2.0382	109	-7
116	Indene	1.6664E-09	-8.7782	2.0639	116	0
146	Indandione	1.3971E-09	-8.8548	2.1895	155	-6
155	2Phenylpyridine	1.4019E-09	-8.8533	2.1871	154	1
161	HMDS	1.3379E-09	-8.8736	2.2203	166	-3
164	tetramethoxythane	1.3153E-09	-8.8810	2.2324	171	-4
178	Diphenylacetylene	1.2020E-09	-8.9201	2.2966	198	-11
204	PhN	1.1174E-09	-8.9518	2.3486	223	-9
304	Tri(o-tolyl)-phosphine	8.4481E-10	-9.0732	2.5477	353	-16
595	Hexaphenyltrisiloxane	6.0717E-10	-9.2167	2.7830	607	-2
	Expanded Discs, ECC_{ED}					
192	9-Methylanthracene	1.2259E-09	-8.9116	2.3107	205	-7
202	Pyrene	1.2195E-09	-8.9138	2.3136	206	-2
228	Triphenylene	1.1129E-09	-8.9535	2.3643	231	-1
433	Tetraphenylnaphthaline	6.2746E-10	-9.2024	2.6819	481	-11
				**Std. dev.**	**σ**	**5**

a) When a compound had more than one signal in the ^{1}H-NMR, the average diffusion coefficient was used.
b) For determining the diffusion coefficient, we used the signal of the $-CH_2$ groups with the highest intensity.

VI. Using Various Molecules as Internal Reference within the same NMR-Sample

S-Table 9: Mixed composition of compounds (each 15 mM) in TOL-d_8 acting them self as reference for the ECC^{TOL}-MW-determination.

		Ref 1 **TOL-d$_7$**		*Ref 2* **ADAM**		*Ref 3* **Si(SiMe$_3$)$_4$**		*Ref 4* **Naphthaline**	
**Analyte**	MW [g/mol]	MW_{det} [g/mol]	ΔMW [%]	MW_{det} [g/mol]	ΔMW [%]	MW_{det} [g/mol]	ΔMW [%]	MW_{det} [g/mol]	ΔMW [%]
TOL-d_7 b)	99	96	3	97	2	96	3	97	2
ADAM a)	136	144	-6	147	-8	144	-6	145	-7
Si(SiMe$_3$)$_4$ a)	321	304	5	309	4	303	5	305	5
Naphthaline b)	128	122	5	124	3	122	5	122	5

a) ECC_{CB}^{TOL}, b) ECC_{DSE}^{TOL} were used to calculate the MW.

VII. Testing the Influence of the Temperature on ECCs

S-Table 10: ECC-MW-determination of Si(SiMe$_3$)$_4$ (321 g/mol) in TOL-d_8 and in THF-d_8. ADAM was used as internal reference in 15mM solutions.

In TOL-d$_8$

**Temp [°C]**	**$D_{x,norm}$ [m/s^2]**	**Log $D_{x,norm}$**	**Log MW_{det}**	**MW_{det} [g/mol]**	**ΔMW [%]**
100	9.5854E-10	-9.0184	2.5113	325	-1
75	9.6025E-10	-9.0176	2.5097	323	-1
50	9.6687E-10	-9.0146	2.5038	319	1
25	9.5261E-10	-9.0211	2.5166	329	-2
0	9.7043E-10	-9.0130	2.5006	317	1
-25	9.5640E-10	-9.0194	2.5132	326	-2
-50	9.9297E-10	-9.0031	2.4807	302	6
-75	9.8113E-10	-9.0083	2.4911	310	3

In THF-d$_8$

**Temp [°C]**	**$D_{x,norm}$ [m/s^2]**	**Log $D_{x,norm}$**	**Log MW_{det}**	**MW_{det} [g/mol]**	**ΔMW [%]**
60	1.0540E-09	-8.9772	2.4975	314	2
45	1.0433E-09	-8.9816	2.5065	321	0
25	1.0671E-09	-8.9718	2.4866	307	4
0	1.0594E-09	-8.9749	2.4930	311	3
-25	1.0766E-09	-8.9680	2.4788	301	6
-50	1.0331E-09	-8.9859	2.5151	327	-2
-75	1.0710E-09	-8.9702	2.4835	304	5

VIII. Gaussian fits of the T1/T2 software of Topspin for LDA in THF-d_8 at 25°C

S-Figure 4: Gaussian fits of the internal reference PhN A) and B). The plots of LDA correspond to α–CH- C) and CH_3 protons D).

a)

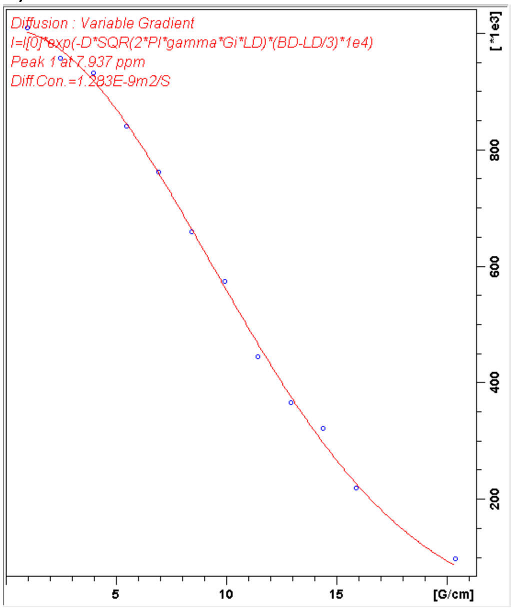

b)

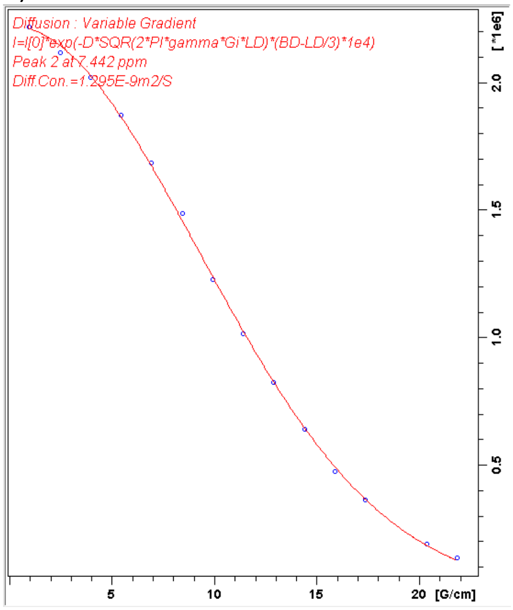

c)

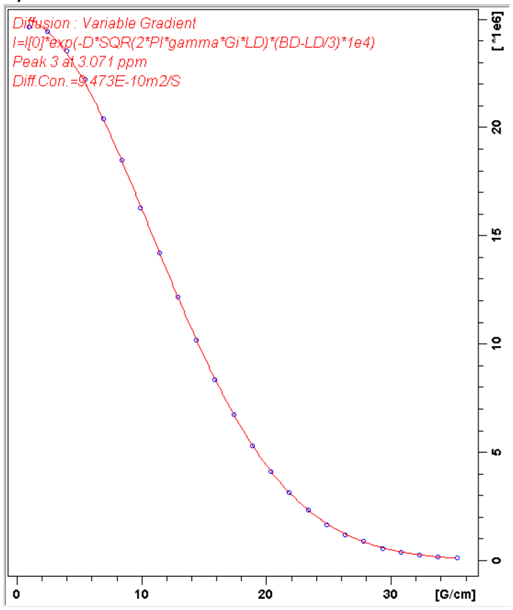

d)

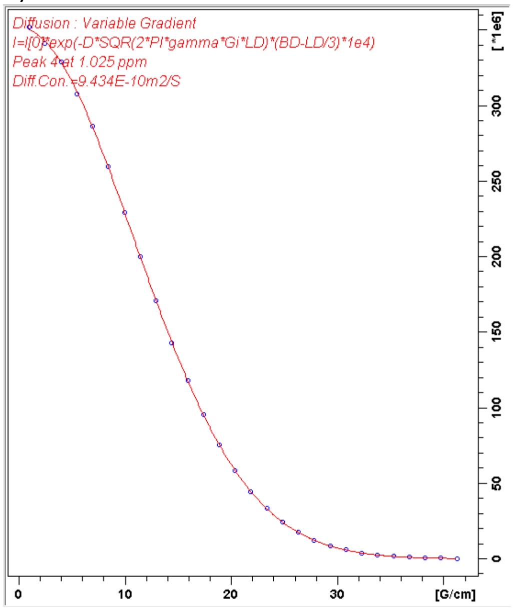

IX. Gaussian fits of the T1/T2 software of Topspin for the $[^tBuLi]_4 \cdot 4[Me_2NC_6H_4Li]_4$ crystal in TOL-d_8 at 25°C

S-Figure 5: ^{7}Li-Dosy of $[^tBuLi]_4 \cdot 4[Me_2NC_6H_4Li]_4$ crystal solvated in TOL-d_8.

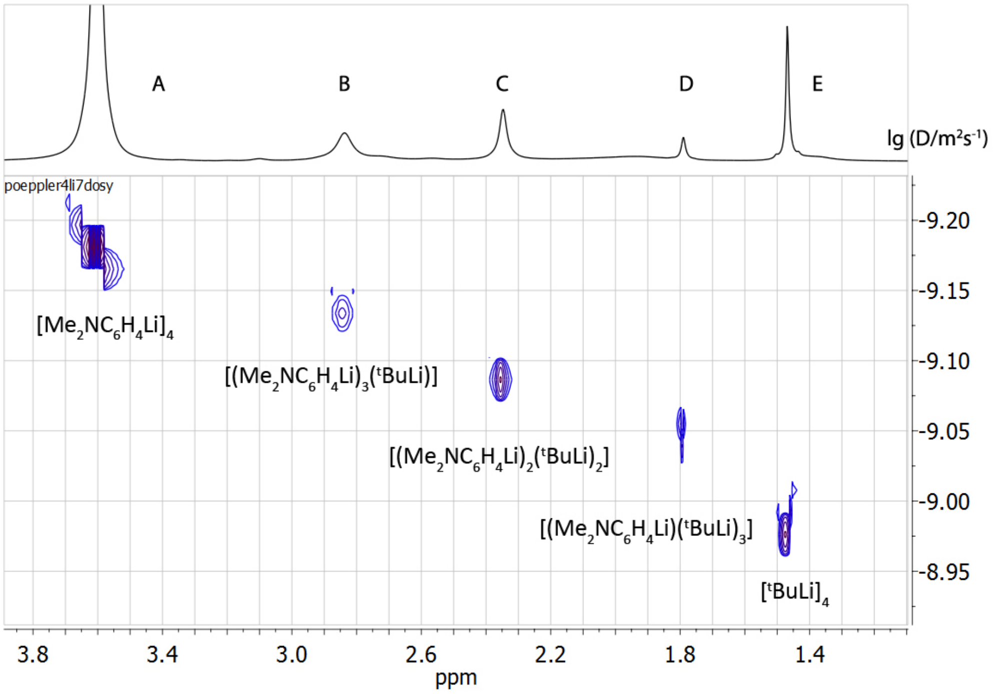

S-Figure 6: Gaussian fits of species A, B, C, D and E. TOL-d_7 was used as internal reference with $\log D_{ref}$ = −8.72.

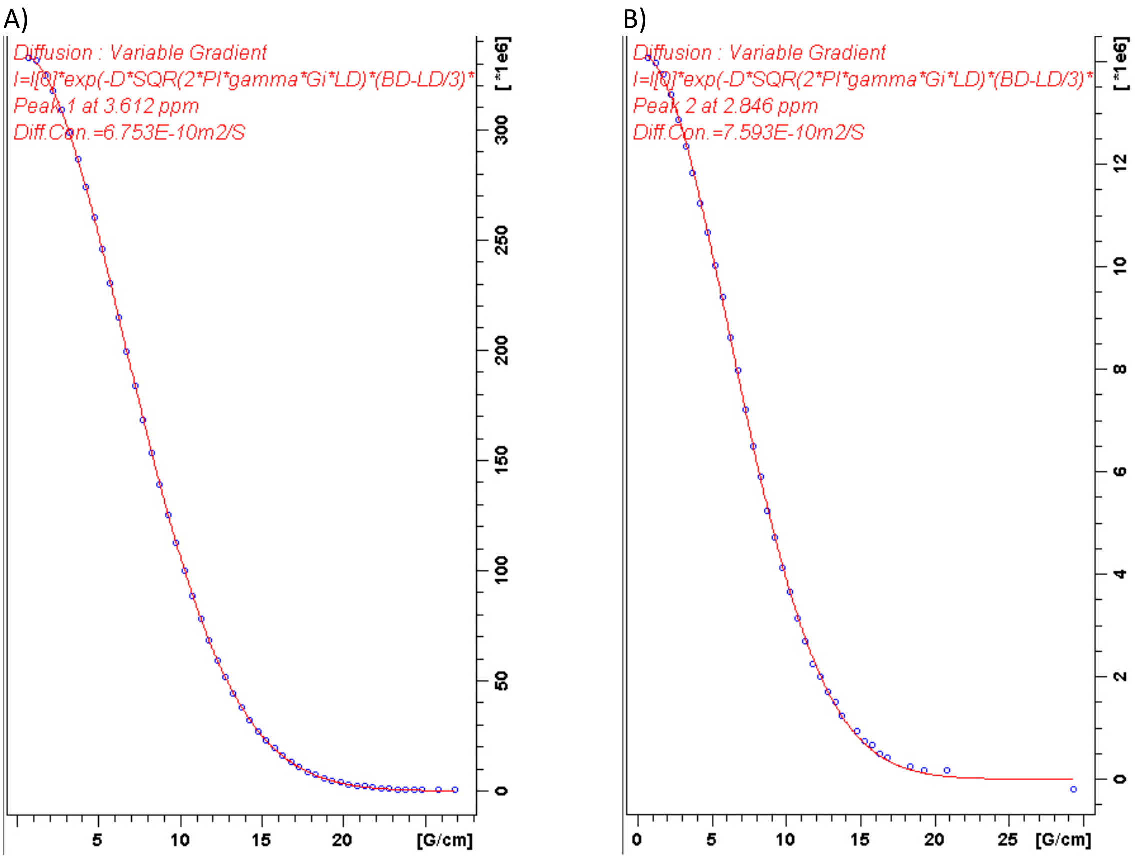

C)

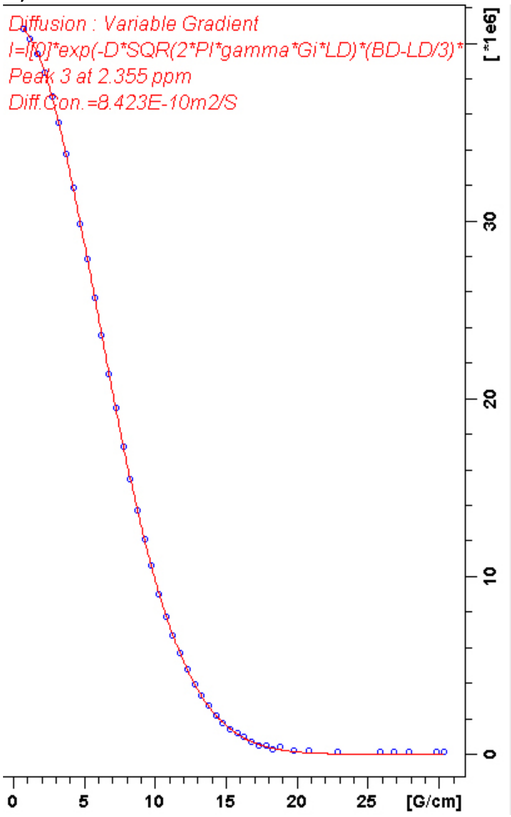
Diffusion : Variable Gradient
I=I[0]*exp(-D*SQR(2*PI*gamma*Gi*LD)*(BD-LD/3)*
Peak 3 at 2.355 ppm
Diff.Con.=8.423E-10m2/S
[G/cm]
[*1e6]

D)

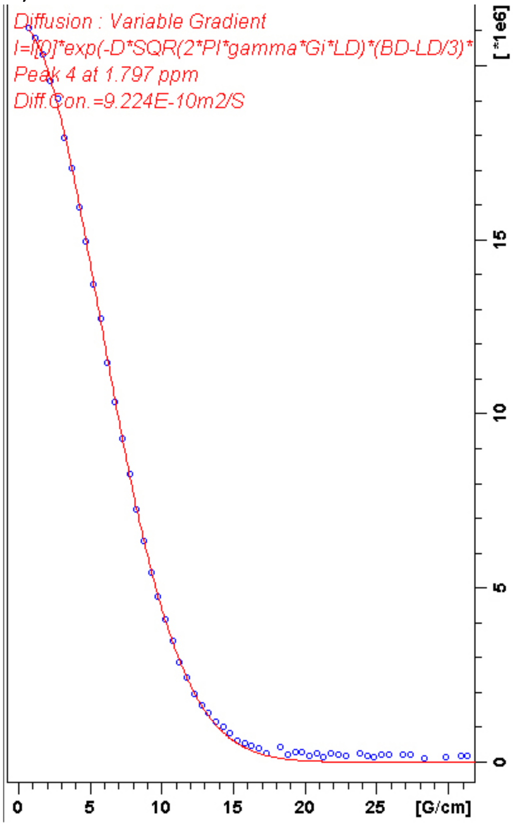
Diffusion : Variable Gradient
I=I[0]*exp(-D*SQR(2*PI*gamma*Gi*LD)*(BD-LD/3)*
Peak 4 at 1.797 ppm
Diff.Con.=9.224E-10m2/S
[G/cm]
[*1e6]

E)

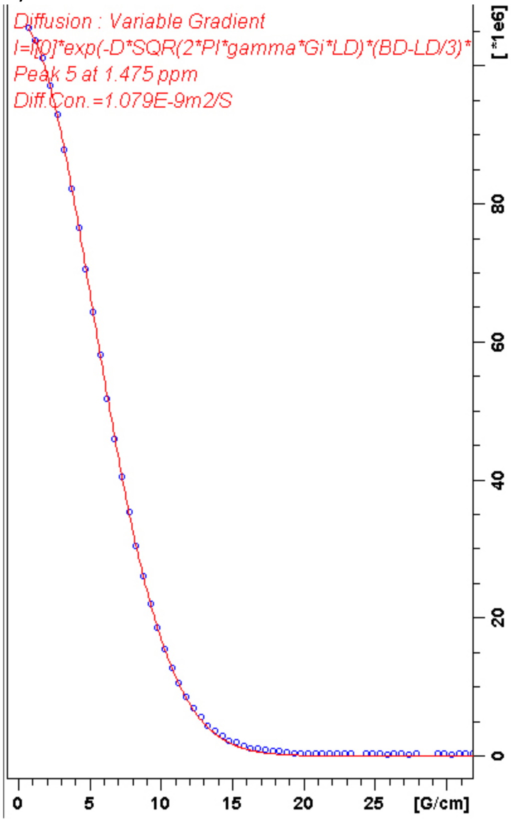
Diffusion : Variable Gradient
I=I[0]*exp(-D*SQR(2*PI*gamma*Gi*LD)*(BD-LD/3)*
Peak 5 at 1.475 ppm
Diff.Con.=1.079E-9m2/S
[G/cm]
[*1e6]

X. Gaussian fits of the T1/T2 software of Topspin for Na-indenide in THF-d_8 at 25°C

S-Figure 7: Gaussian fits of A, B) Na-indenide, C) indene, D) TMB and E) (H)HMDS

A)

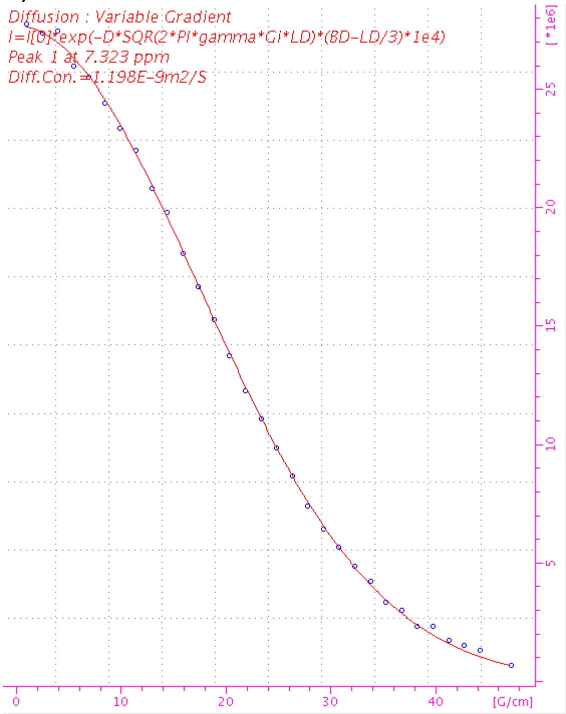

B)

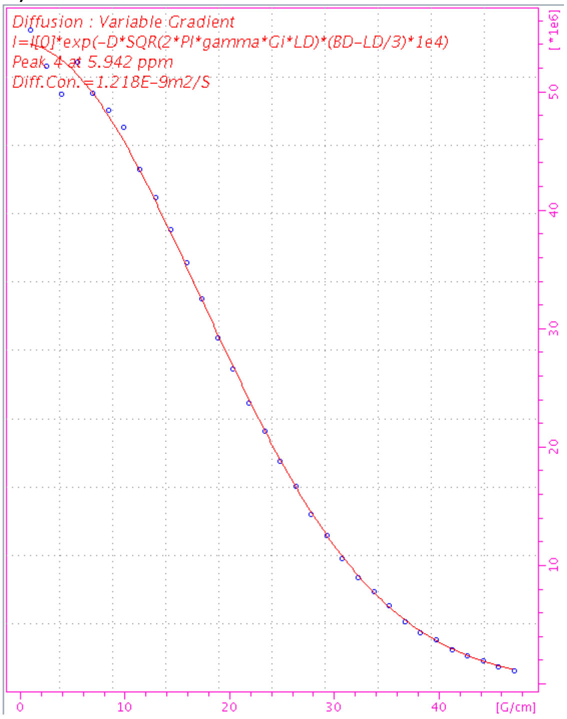

C)

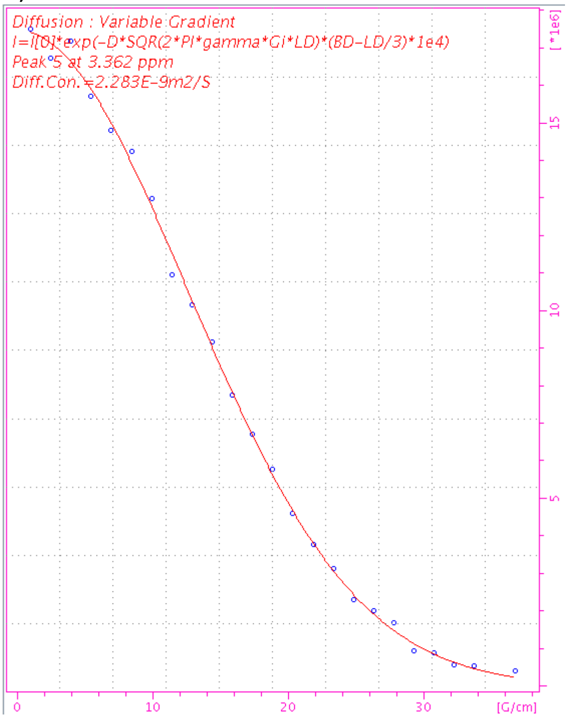

D)

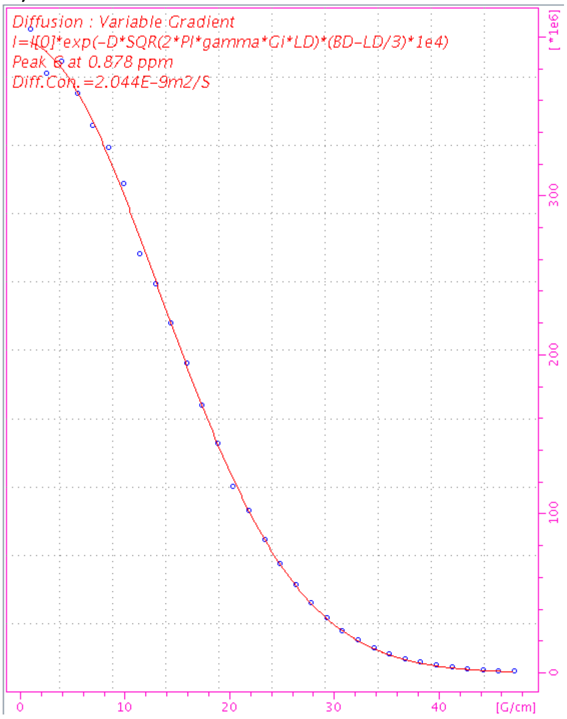

E)

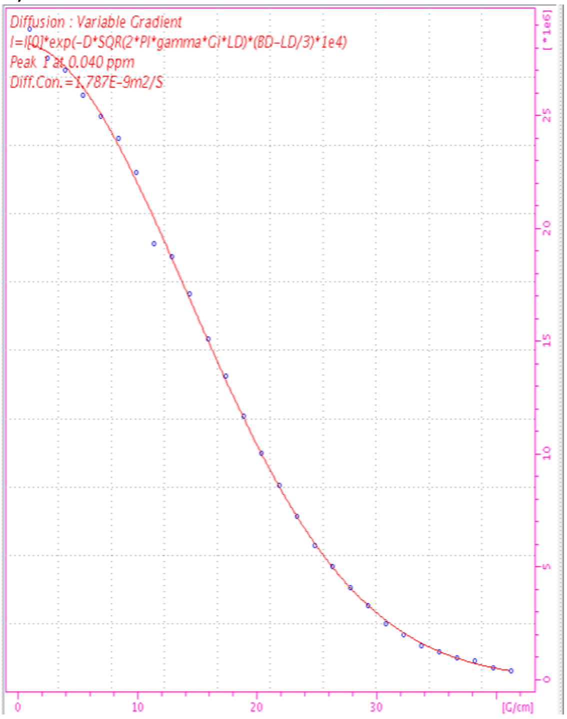

XI. Gaussian fits of the T1/T2 software of Topspin for Na-indenide in THF-d_8 at -50°C

S-Figure 8: Gaussian fits of A, B) Na-indenide, C) indene, D) TMB and E) (H)HMDS

A)

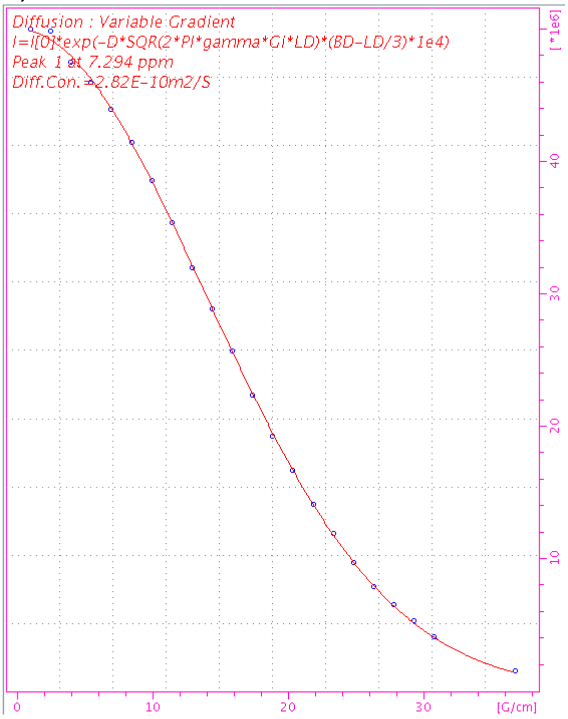

B)

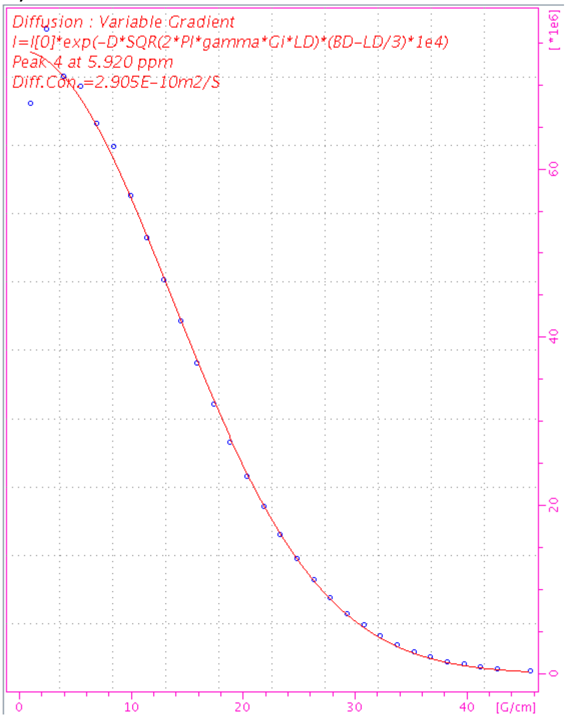

C)

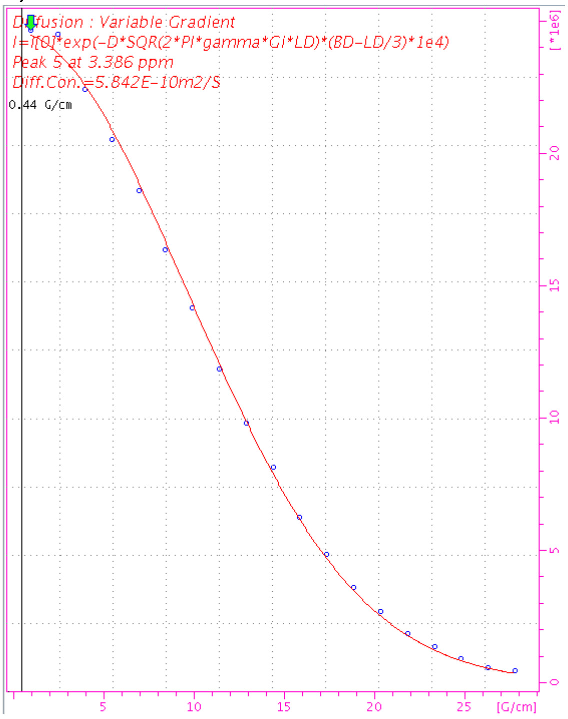
Diffusion : Variable Gradient
I=I[0]*exp(-D*SQR(2*PI*gamma*Gi*LD)*(BD-LD/3)*1e4)
Peak 5 at 3.386 ppm
Diff.Con.=5.842E-10m2/S
0.44 G/cm
[G/cm]
[*1e6]

D)

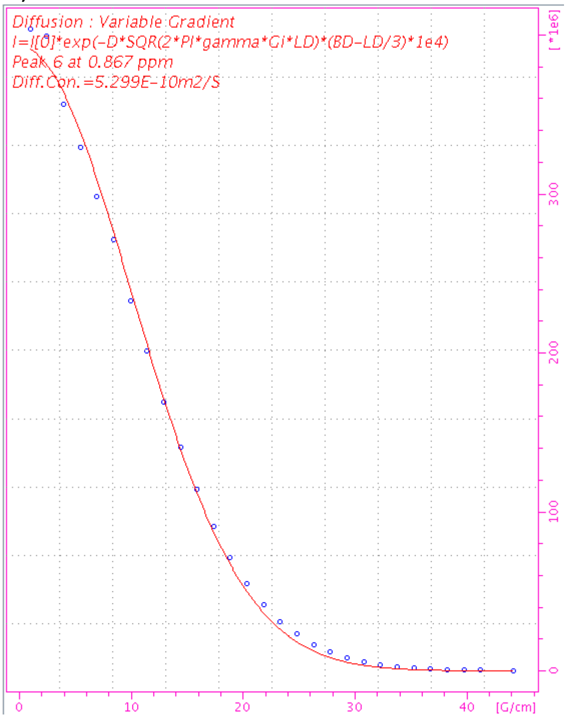
Diffusion : Variable Gradient
I=I[0]*exp(-D*SQR(2*PI*gamma*Gi*LD)*(BD-LD/3)*1e4)
Peak 6 at 0.867 ppm
Diff.Con.=5.299E-10m2/S
[G/cm]
[*1e6]

E)

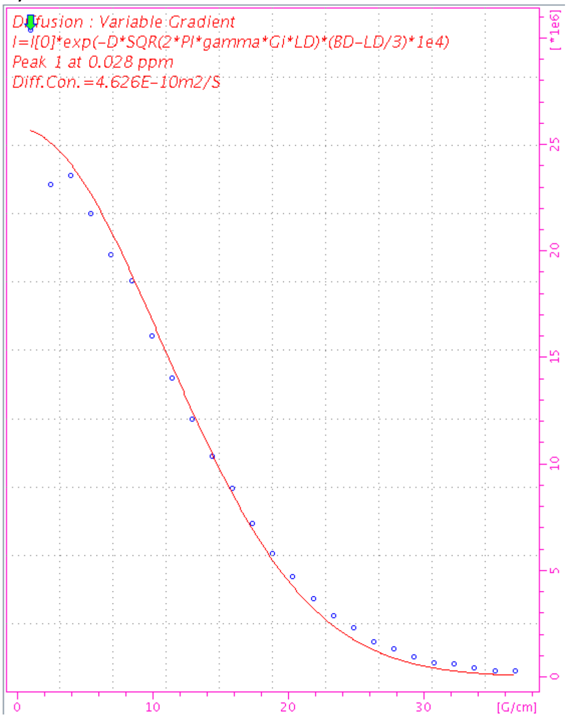
Diffusion : Variable Gradient
I=I[0]*exp(-D*SQR(2*PI*gamma*Gi*LD)*(BD-LD/3)*1e4)
Peak 1 at 0.028 ppm
Diff.Con.=4.626E-10m2/S
[G/cm]
[*1e6]

XII. Gaussian fits of the T1/T2 software of Topspin for Na-indenide in THF-d_8 at 60°C

S-Figure 9: Gaussian fits of A, B) Na-indenide, C) indene, D) TMB and E) (H)HMDS

A)

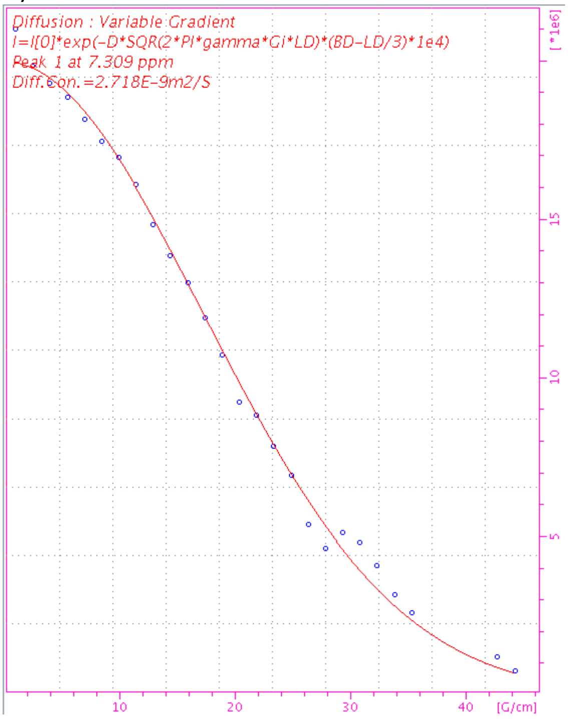

B)

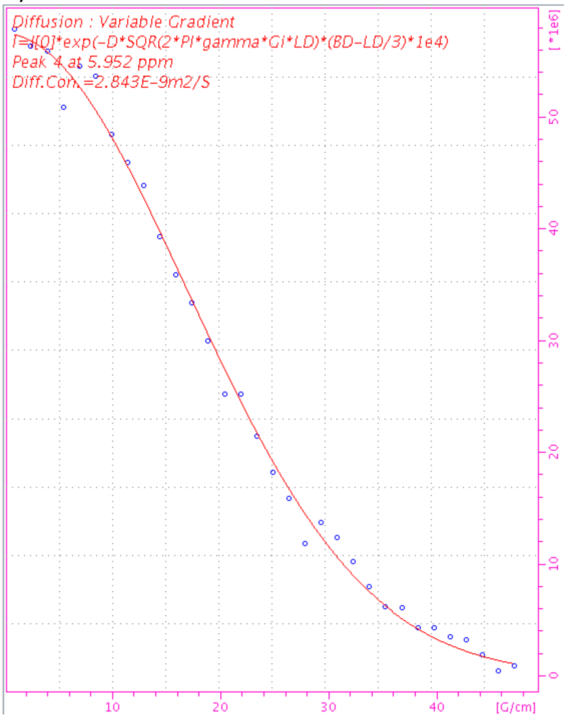

C)

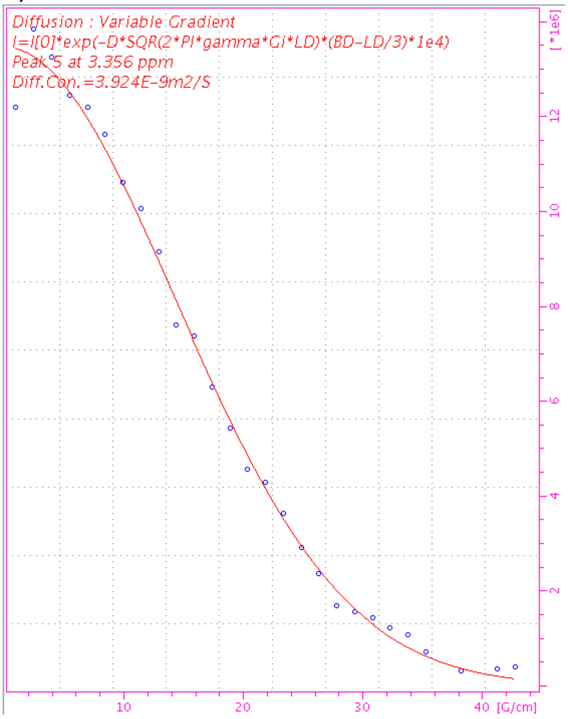

D)

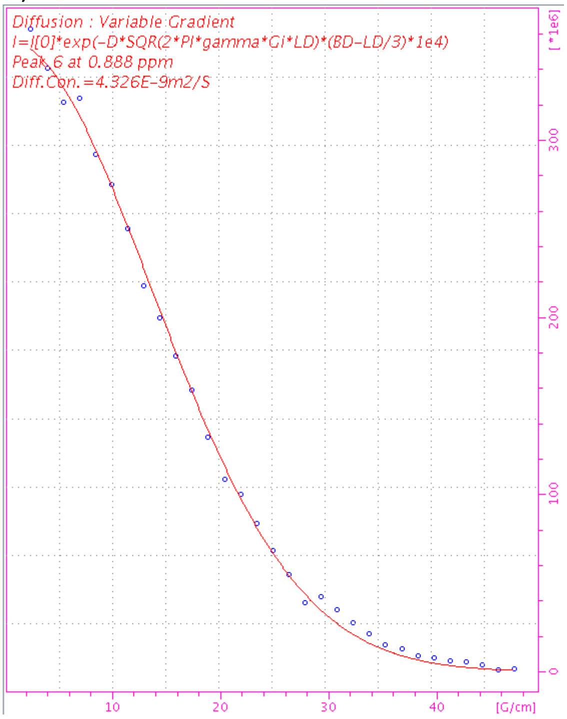

E)

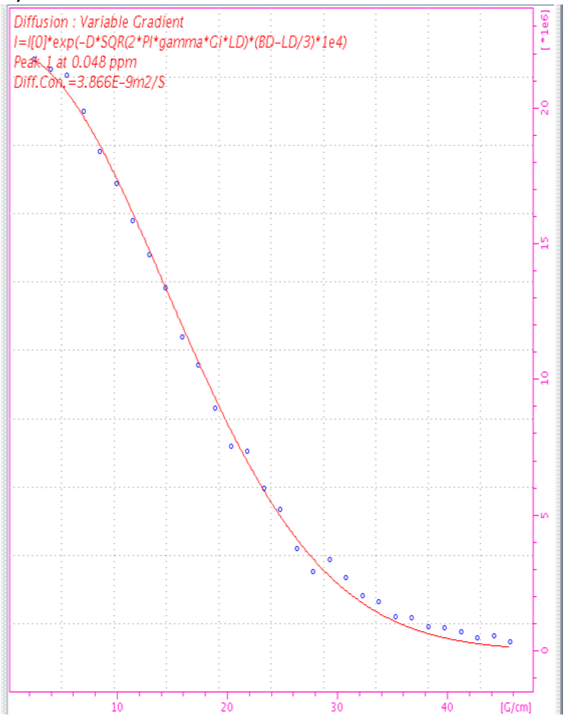

Diffusion : Variable Gradient
I=I[0]*exp(-D*SQR(2*PI*gamma*Gi*LD)*(BD-LD/3)*1e4)
Peak 1 at 0.048 ppm
Diff.Con.=3.866E-9m2/S
[*1e6]
20
15
10
5
0
10
20
30
40
[G/cm]

XIII. Calculation of the Molar Van-der-Vaals Density MD_w

S-Table 11: Calculation of the molar Van-der-Waals density *MD* using equation (**S5**)

Compound	Formula	C	H	N	O	Si	P	S	Cl	Br	Na	*MW* [g/mol]	MD_w [g/(mol · m³)]	ΣV_W [m³]
Cyclopentane	C5H10	5	10	--	--	--	--	--	--	--	--	70	4.41E+29	1.59E-28
THF	C4H8O	4	8	--	1	--	--	--	--	--	--	72	5.08E+29	1.42E-28
TMS	C4H12Si	4	12	--	--	1	--	--	--	--	--	88	4.68E+29	1.88E-28
MTBE	C5H12O	5	12	--	1	--	--	--	--	--	--	88	4.77E+29	1.85E-28
TMB	C8H18	8	18	--	--	--	--	--	--	--	--	114	4.30E+29	2.65E-28
ADAM	C10H16	10	16	--	--	--	--	--	--	--	--	136	4.61E+29	2.95E-28
$N(SiMe_3)_3$	C9H27NSi3	9	27	1	--	3	--	--	--	--	--	234	5.00E+29	4.68E-28
$Si(SiMe_3)_4$	C12H36Si5	12	36	--	--	5	--	--	--	--	--	321	5.00E+29	6.42E-28
Toluol	C7H8	7	8	--	--	--	--	--	--	--	--	92	4.88E+29	1.89E-28
Diisopropylether	C6H14O	6	14	--	1	--	--	--	--	--	--	102	4.72E+29	2.16E-28
Indene	C9H8	9	8	--	--	--	--	--	--	--	--	116	5.05E+29	2.30E-28
Naphthaline	C10H8	10	8	--	--	--	--	--	--	--	--	128	5.11E+29	2.50E-28
1,3 Indandione	C9H6O2	9	6	--	2	--	--	--	--	--	--	146	5.89E+29	2.48E-28
2-Phenylpyridine	C11H9N	11	9	1	--	--	--	--	--	--	--	155	5.31E+29	2.92E-28
Tetramethoxypropane	C7H16O4	7	16	--	4	--	--	--	--	--	--	164	5.61E+29	2.92E-28
Diphenylacetylene	C14H10	14	10	--	--	--	--	--	--	--	--	178	5.18E+29	3.44E-28
Diphenylsulfoxid	C12H10OS	12	10	--	1	--	--	1	--	--	--	202	5.91E+29	3.42E-28
1-Phenylnaphthaline	C16H12	16	12	--	--	--	--	--	--	--	--	204	5.15E+29	3.96E-28
Tri(o-tolyl)-phosphine	C21H21P	21	21	--	--	--	1	--	--	--	--	304	5.30E+29	5.74E-28
Hexaphenyltrisiloxane	C36H30O3Si3	36	30	--	3	3	--	--	--	--	--	595	5.57E+29	1.07E-27
BINAP	C44H32P2	44	32	--	--	--	2	--	--	--	--	623	5.50E+29	1.13E-27
Anthracene	C14H10	14	10	--	--	--	--	--	--	--	--	178	5.18E+29	3.44E-28
Acridine	C13H9N	13	9	1	--	--	--	--	--	--	--	179	5.37E+29	3.33E-28
9-Methylanthracene	C15H12	15	12	--	--	--	--	--	--	--	--	192	5.11E+29	3.76E-28
Pyrene	C16H10	16	10	--	--	--	--	--	--	--	--	202	5.25E+29	3.85E-28
Anthrachinone	C14H8O2	14	8	--	2	--	--	--	--	--	--	208	5.74E+29	3.62E-28
Triphenylene	C18H12	18	12	--	--	--	--	--	--	--	--	228	5.21E+29	4.37E-28
Tetraphenylnaphthaline	C34H24	34	24	--	--	--	--	--	--	--	--	433	5.19E+29	8.34E-28
1-Hexylchloride	C6H13Cl	6	13	--	--	--	--	--	1	--	--	120	5.49E+29	2.18E-28
1-Octylchloride	C8H17Cl	8	17	--	--	--	--	--	1	--	--	149	5.29E+29	2.82E-28
1-Decylchloride	C10H21Cl	10	21	--	--	--	--	--	1	--	--	177	5.13E+29	3.45E-28
1-Propylbromide	C3H7Br	3	7	--	--	--	--	--	--	1	--	123	9.66E+29	1.27E-28
Dibromoanthracene	C14H8Br2	14	8	--	--	--	--	--	--	2	--	336	8.71E+29	3.86E-28
Triphenylmethylbromid	C19H15Br	19	15	--	--	--	--	--	--	1	--	323	6.45E+29	5.01E-28
M1	C13H15NaO	13	15	--	1	--	--	--	--	--	1	218	5.25E+29	4.15E-28
M2	C17H23NaO2	17	23	--	2	--	--	--	--	--	1	298	5.35E+29	5.56E-28
M3	C21H31NaO3	21	31	--	3	--	--	--	--	--	1	379	5.43E+29	6.98E-28
M4	C21H31NaO3	25	39	--	4	--	--	--	--	--	1	426	5.07E+29	8.40E-28
D1	C26H30Na2O2	26	30	--	2	--	--	--	--	--	2	437	5.27E+29	8.30E-28
D2	C34H46Na2O4	34	46	--	4	--	--	--	--	--	2	597	5.36E+29	1.11E-27

$$MD_W = \frac{MW}{\sum V_W} = MW \Big/ \sum_{i=1}^{n} \frac{3}{4}\pi r_{w,i}^{3} \qquad \textbf{(S5)}$$

where MD_W is the molar Van-der-Waals density, *MW* the molecular weight, V_W the Van-der-Waals volume and r_W the Van-der-Waals radius.

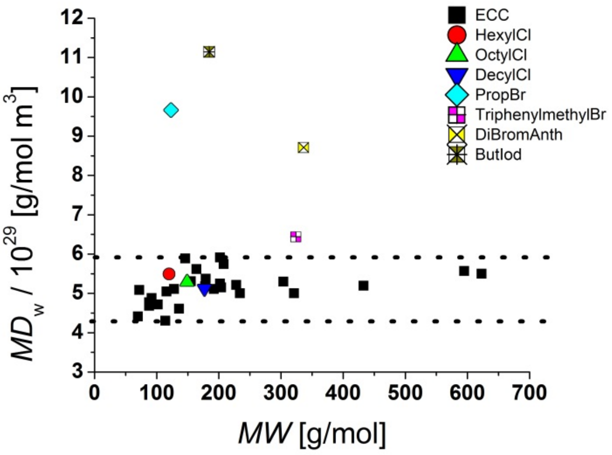

S-Figure 10: Weight distribution in the model compounds and molecules with heavy atoms.

Molecules with a molar density between $4.3\cdot10^{29}$ g/(mol · m^3) and $5.9.0\cdot10^{29}$ g/(mol · m^3) work well with the ECCs that are presented in this article.

S-Table 12: Van-der-Waals Volumes of selected atoms.

**Atom**	**V_W [a)] [m³]**
H	5.575E-30
C	2.058E-29
N	1.560E-29
O	1.471E-29
Na	4.900E-29
Si	3.880E-29
P	2.443E-29
S	2.443E-29
Cl	2.245E-29
Br	2.652E-29
I	3.252E-29

[a)] The Van-der-Waals radii were taken from A. Bondi, *J. Phys. Chem*. **1964**, *68*, 441-451.

XIV. ECC-MW-Determination Excel Spreadsheet

A simple Excel spreadsheet is available at

http://www.stalke.chemie.uni-goettingen.de/mw_det_calc/mw_det_calc.xlsx

That implements the calculation of log$D_{x,norm}$ described in the main text, allowing to estimate MWs of analytes from their diffusion coefficients. Please read the information on the first excel sheet.
